# Breeding colored sweet corn for improved micronutrient content

**DOI:** 10.3389/fpls.2026.1813937

**Published:** 2026-05-18

**Authors:** Jonathan Niyorukundo, Caleb Wehrbein, Anne Fischer, Sophie Alvarez, David R. Holding

**Affiliations:** 1 Department of Agronomy and Horticulture, University of Nebraska, Lincoln, NE, United States; 2 Proteomics & Metabolomics Facility, Center for Biotechnology, University of Nebraska, Lincoln, NE, United States

**Keywords:** anthocyanins, carotenoids, flavonoids, kernel color, maize pigments, sweet corn

## Abstract

Sweet corn is mainly grown for human consumption as a fresh, frozen, and canned vegetable and it is valued for its sweetness, flavor, tenderness, and antioxidant properties associated with the carotenoid pigments found in yellow kernels. However, sweet corn lacks diverse kernel color pigmentation often found in field corn as colors often accumulate after the prime eating stage. There is nutritional and market potential, in terms of micronutrients and aesthetic diversity, for creating eating-stage sweet corn with various colored flavonoid compounds. This study sought to generate *sugary-1(su1)* and *shrunken-2(sh2)* sweet corn hybrids that accumulated beneficial pigmentation by the prime sweet corn eating stage. Various colored maize varieties of dent corn, and flint corn types were crossed to eight sweet corn inbreds bidirectionally. This was followed by five selfing generations while selecting for color, sweetness, and texture phenotypes at prime-eating stage (20 days after pollination; DAP). Nine colored sweet corn (CS) inbreds were selected and were used to produce 20 CS hybrids. The CS lines were selected for their satisfactory sweetness, flavor, and texture phenotypes. Yellow-orange, purple, blue, and red were the predominant kernel colors observed in the selected inbreds and these color continued to manifest in the hybrids. CS inbreds and hybrids had maintained kernel sweetness and tenderness comparable to parental sweet corns. Analysis of the carotenoids and flavonoids revealed a dynamic increase and decrease of pigment concentrations among the CS inbreds in comparison with the parental sweet corn inbreds. Biochemical assays confirmed that the CS sugar and starch contents did not change substantially from the normal ranges reported in sweet corn parents. This study demonstrates that diverse colors readily accumulate by prime eating stage in sweet corn and thus increase sweet corn carotenoid and flavonoid profiles and the potential to improve its nutritional quality and market potential.

## Introduction

Sweet corn is widely grown vegetable world-wide. Unlike other maize types, sweet corn is harvested at its prime eating stage of approximately 20 DAP (Lertrat and Pulam, 2007). It is highly valued for its high sucrose to starch ratio, tenderness and flavors. For decades, sweet corn breeding efforts have focused primarily on maximizing the sweetness, sensory performance and eating quality (Branch et al., 2024 and Jain et al., 2006). This might have resulted in less exploration of other quality and specialty traits that may be beneficial to the overall end use quality of sweet corn. Color diversity has not been a priority in sweet corn breeding. Unlike other maize subspecies bred for wide ranging kernel colors, color in sweet corn may have been lost during artificial selection by early humans resulting in yellow and white sweet corn being globally adopted (Hu et al., 2016; Hong et al., 2020). Maize color diversity reflects the presence of carotenoid, flavonoid, and other color-associated pigments that are shown to confer numerous nutritional and health benefits. Maize kernel color and the associated nutrition and health benefits are valuable drivers of consumer preferences (LaPorte et al., 2022). Consequently, increasing sweet corn color diversity may improve sweet corn nutritional and market potential.

Maize kernel pigmentation is attributed to the accumulation of compounds produced by the secondary metabolites, carotenoids and flavonoids (Chatham et al., 2019). The carotenoids fall into two groups based on their functions and structure: carotenes, including a- and b-carotenes, and xanthophylls, like b-cryptoxanthin, zeaxanthin, and lutein (Nabi et al., 2020). The carotenoids are responsible for the formation of colors ranging from yellow to orange (Rodríguez et al., 2013; Chatham et al., 2019), and they possess health benefits due their antioxidant, anti-inflammatory, antibacterial, and anti-cancer properties (Nabi et al., 2020). The carotenoid pigments like b-carotene serve as the precursor to vitamin A, and the yellow pigment, zeaxanthin slows down the age-related macular eye disease and cataracts (O’Hare et al., 2015; Eggersdorfer and Wyss, 2018). Apart from the carotenoids, the flavonoids, mainly the anthocyanins contribute to a wide range of kernel colors in maize. The flavonoids are categorized into classes such as anthocyanins, flavanols, chalcones, and flavanones (Panche et al., 2016; Alappat and Alappat, 2020). They are responsible for colors ranging from red to orange, blue, and purple (Alappat and Alappat, 2020). The flavonoids possess various health promoting benefits because of their antioxidative, anti-inflammatory, and anti-carcinogenic properties (Abdel-Aal et al., 2006; Panche et al., 2016). Given numerous nutrition and health benefits associated with the carotenoids and the flavonoids, effort to introduce them into common sweet corn varieties may expand the demand and potential for colored sweet corn varieties.

Despite the potential health benefits of these maize kernel pigmentations, breeding and maintaining diverse colors into sweet corn has proven challenging. Maize kernel pigmentations fully develop at the kernel physiological maturity. This timing conflicts with the fact that sweet corn must be harvested and consumed or processed at its milk stage (Lago et al., 2014; O’Hare et al., 2015). For instance, it was reported that the concentration of anthocyanin increased significantly with the kernel maturity and delayed harvesting were favorable for more accumulation (Hong et al., 2020). In addition, it was also found that zeaxanthin increased with kernel maturity (O’Hare et al., 2015). Moreover, the sugar and moisture contents are highest at this stage and subsequently, sugar to starch conversion is accelerated which substantially reduces the sweetness and eating quality (Hong et al., 2020). Sweet corn exhibits endospermic mutations such as *sugary-1* and *shrunken-2* which increase the sugar to starch ratio in the endosperm by perturbing different parts of the starch biosynthesis pathway (Hu et al., 2021). Since kernel carotenoid and flavonoid pigments accumulate in the inner starchy endosperm, and the aleurone endosperm layer respectively (Ford, 2000), the reduction of starch accumulation, due to the *su1* or *sh2* mutations at sweet corn’s prime-eating stage, might affect the accumulation of these pigments. In a study conducted by Branch et al. (2024), they reported that in yellow maize, the presence of *sugary enhancer1(se1)* was correlated with the reduced levels of lutein and zeaxanthin compared to the *Se1* counterpart. Similarly, another study showed that the *sh2* gene mutation responsible for the high sweetness trait of sweet corn is tightly linked (<0.1 cM) to the non-functional mutated *A1* gene responsible for the purple pigment in maize (Anirban and O'Hare, 2020; Anirban et al., 2023). The *A1* gene encodes for dihydroflavonol 4-reductaase that makes dihydroflavonol, a precursor to 3-deoxyanthocyanin responsible for various pigments in maize and sorghum (Bernhardt et al., 1998; Herrman et al., 2020). The challenge to conventional breeding approaches such as the one used in this study, lies with breaking the linkage in order to crossover the *A1* allele with the *sh2* allele using the traditional breeding method. Anirban et al. (2023) developed a purple sweet corn using a conventional breeding technique despite the challenge of breaking the linkage between *sh2* and *a1*. Previous maize pigments breeding efforts focused on introgessing specific kernel pigment and individual carotenoid and flavonoid pigments into *sh2* sweet corns. Here, we sought to breed diverse maize pigments primarily into *sugary-1(su1)* sweet corn inbreds using conventional breeding approach. We used diversely pigmented maize lines of flint corn, dent corn, and popcorn types to explore their diverse kernel color phenotypes in su1 sweet corn. A *sh2* sweet corn inbred was also used.

The objective of this study was first, to establish that significant pigment can accumulate by the prime eating stage. We then sought to develop diversely colored sweet corn hybrids of *su1* type and explore how they compare among themselves and the *sh2* colored sweet corn hybrids. Eight parental sweet corn inbreds were crossed to different colored maize lines, followed by five rounds of self-pollination and selection. Since the colored maize lines used had high starch content compared to sweet corn, the selection prioritized the quality traits such as sweetness, and kernel tender texture and flavor. Nine colored sweet corn inbreds were developed and were used to produce 20 colored sweet corn hybrids. Sweet corn lines were arbitrarily scored for their quality traits, analyzed for their sugar and starch contents, and profiled for their carotenoid and flavonoid compounds. The results indicated that the colored sweet corn inbreds and hybrids exhibited kernel sweetness, texture, and flavors similar to the parental sweet corns. Sugar and starch contents did not differ significantly from the normal levels of parental sweet corns whereas the carotenoids and flavonoids were increased in some colored sweet corn genotypes. This is a preliminary proof of concept study, and the identified superior genotypes will be next be subjected to multilocation field trials.

## Materials and methods

### Germplasm

Seeds were acquired from the public seed stock centers and multiple seed suppliers including Mary’s Heirloom seeds, Sherwood’s seeds, and Baker Creek Heirloom seeds. The experimental seed material consisted of eight sweet corn varieties and numerous varieties of colored maize such as dent corn, and flint corn. (**Supplementary Table 1**). The sweet corn parental materials (designated S1-S8) consisted of NE-EDR *su1*, NE-EDR *sh2*, P39-*su1*, IA5125-*su1*, IA453-*su1*, P51-*su1*, FL32 *su1* and *sh2*, and FL56 *su1* and *sh2* respectively while the colored maize varieties included Bloody Butcher, Rainbow Flint Red, Blue Indian, Coburn’s Early Red, and Glass Gem. Additional colored maize including Double Red Sweet Corn and Seneca Red Stalker were used in cases where color was weak in the first introgression.

### Field design

The field experiments were conducted at the University of Nebraska-Lincoln East Campus Research fields in Lincoln, Nebraska (40.83486°N, 96.66377°W). The field was rain-irrigated in the Summer of 2020–2022 with supplemental drip-irrigation utilized in the summer of 2023 and 2024. During the initial evaluation and preselection phase (2020-2021), genotypes were planted in a single row and unreplicated to facilitate early selection. Then, from 2023 forward, genotypes were planted in a Completely Randomized Design (CRD) where they were organized in 14 m^2^ blocks and a 60 cm buffer alley separating adjacent blocks. To minimize the damage of wild animals, an electric fence was installed around the field followed by a four-row field corn border.

### Production and selection of colored sweet corn inbreds and hybrids

**Figure 1:**
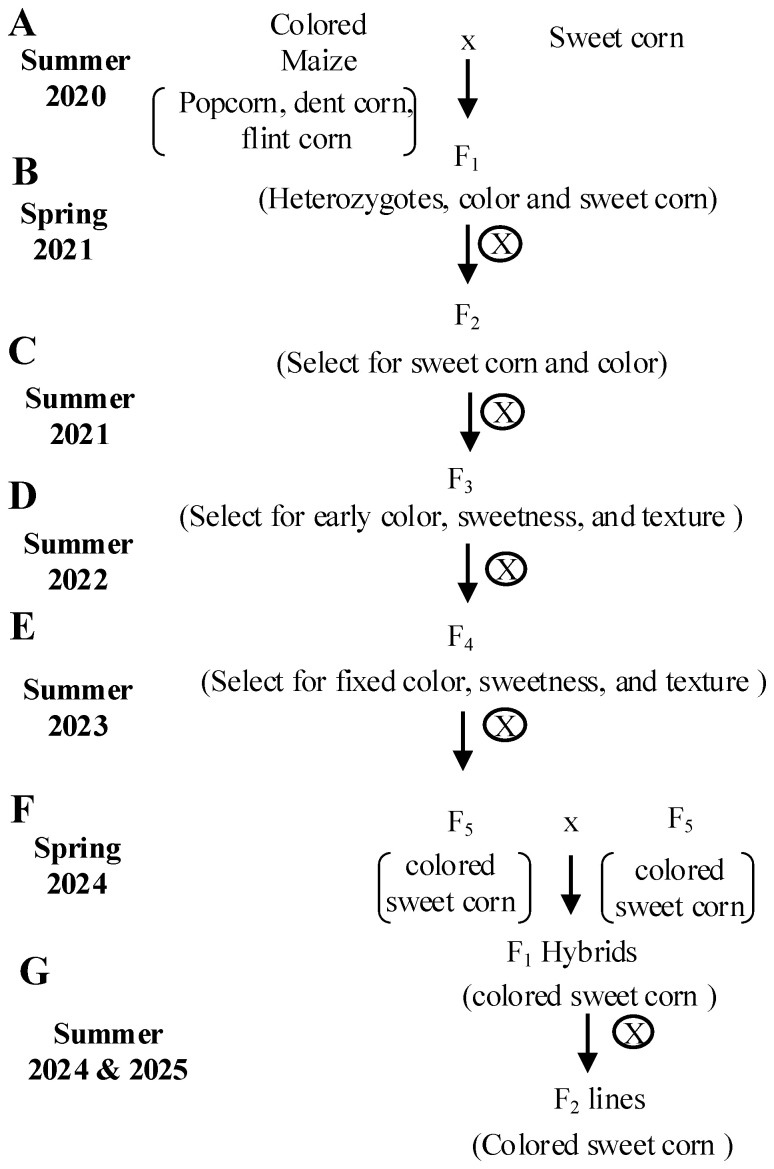
Breeding scheme for the development of colored sweet corn inbreds. **(A)** F_1_ crosses were made bidirectionally between colored maize and *su1* or *sh2* sweet corn lines, using both as male and female parents. **(B)** F_2_ plants with desirable agronomic traits were self-pollinated to produce F_2_. **(C)** F_₂_ populations segregated for kernel color and sweetness; visually selected colored F_2_ kernels were self-pollinated to obtain F_3_. **(D)** F_3_ ears were evaluated for color, taste, and texture at 20 days after pollination (DAP). Sensory traits were scored from 1 (poor) to 5 (excellent); top-performing lines were self-pollinated to generate F_4_. **(E)** F_4_ lines were evaluated similarly; only lines scoring ≥3 in all traits and with desirable agronomic characteristics were advanced. F_4_ plants were self-pollinated to produce F_5_ inbreds. **(F)** Top-performing inbreds were selected based on uniform color, sweetness, and texture and were used to produce F_1_ hybrids via bidirectional crosses. **(G)** F_2_ lines were obtained by selfing F_1_s and subjected to sensory evaluation to identify superior-colored sweet corn hybrids.

The parental sweet corn and colored maize varieties were bulked first, and distinct combination crosses were made. This was followed by phenotypic selection at each generation for vivid kernel color pigmentation, optimum sugar and starch content, and good plant and seed agronomics (**Figure 1**). During the summer of 2020, initial crosses were made between the sweet corn parents and different colored maize parents. In the spring of 2021, the F_1_ heterozygotes seeds were self-pollinated in the greenhouse to produce F_2_ ears segregating for the classical mature kernel sweet corn phenotypes as well as variable spectrums of color. Mature F_2_ kernels with shrunken sweet corn and color phenotypes were selected and, in the summer of 2021, they were grown and self-pollinated in the field to produce F_3_ colored sweet corn lines. Kernel color phenotypes were visually verified in mature F_3_ lines and were selected for the next generation. F_3_ seeds were self-pollinated in the field for two successive generations (summer of 2022 and 2023) to produce fixed color F_5_ sweet corn lines, Colored Sweet corn (CS) inbreds (**Table 1**). In the spring of 2024, F_1_ hybrids (**Table 2**) were made between colored sweet corn inbreds in the greenhouse. Parents for hybrids were selected on the basis of uniform color where possible and good sweetness, tenderness and flavor. A single location was used for preliminary testing of all hybrids in 2024 and 2025. Later, multiple location trials will be conducted on the best hybrids identified in the current study. In the summer of 2024 and 2025, F_1_ CS hybrids were grown and were self-pollinated to produce F_2_ CS lines that were used for evaluating the F_1_ hybrids.

**Table 1:** Pedigree details for the development of F_5_ CS inbreds.

Ref. no.	Pedigree
CS1	(NE-EDR su1/Blue Indian) → F_1B_ → F_2B_ → F_3B_ → F_4B_ → F_5_
CS2	(NE-EDR sh2/Blue Indian) → F_1B_ → F_2B_ → F_3B_ → F_4B_ → F_5_
CS3	(P39 su1/Blue Indian) → F_1B_ → F_2B_ → F_3B_ → F_4B_ → F_5_
CS4	(IA5125 su1/Bloody Butcher) → F_1B_ → F_2B_ → F_3B_ → F_4B_ → F_5_
CS5	((NE-EDRsh2/Blue Indian) F_2_//Seneca Red Stalker) → F_1B_ → F_2B_ → F_3B_ → F_4B_ → F_5_
CS6	(Double Red Sweet corn//(NE-EDR su1/Blue Indian) F_2_) → F_1B_ → F_2B_ → F_3B_ → F_4B_ → F_5_
CS7	((NE-EDR sh2/Blue Indian) F2//Double Red Sweet corn) → F_1B_ → F_2B_ → F_3B_ → F_4B_ → F_5_
CS8	(NE-EDR sh2/Bloody Butcher) → F_1B_ → F_2B_ → F_3B_ → F_4B_ → F_5_
CS9	(NE-EDR su1/Bloody Butcher) → F_1B_ → F_2B_ → F_3B_ → F_4B_ → F_5_

Nine final CS inbreds were generated. Inbreds were selected for color trait and sweet corn eating qualities. “B” stands for bulking. For simplicity, reference numbers CS1-CS9 were used for identification of inbreds in this analysis. Sweet corn parents were referenced as S1: IA5125su1, S2:IA453su1, S3:P39su1, S4:P51su1, S5: NE-EDRsu1, S6:NE-EDRsh2.

**Table 2:** Pedigree details for the development of F_1_ CS hybrids.

Ref. no.	Crosses	Pedigree
H1	CS1+CS3	(NE-EDR su1/Blue Indian) F_5_ + (P39 su1/Blue Indian) F_5_
H2	CS1+CS6	(NE-EDR su1/Blue Indian) F_5_ + ((Double Red Sweet corn//(NE-EDR su1/Blue Indian) F_2_) F_5_
H3	CS1+CS8	(NE-EDR su1/Blue Indian) F_5_ + (NE-EDR sh2/Bloody Butcher) F_5_
H4	CS1+CS9	(NE-EDR su1/Blue Indian) F_5_ + (NE-EDR su1/Bloody Butcher) F_5_
H5	CS2+CS4	(NE-EDR sh2/Blue Indian) F_5_ + (IA5125 su1/Bloody Butcher) F_5_
H6	CS2+CS6	(NE-EDR sh2/Blue Indian) F_5_ + ((Double Red Sweet corn//(NE-EDR su1/Blue Indian) F_2_) F_5_
H7	CS2+CS7	(NE-EDR sh2/Blue Indian) F_5_ + ((NE-EDR sh2/Blue Indian) F2//Double Red Sweet corn)) F_5_
H8	CS2+CS8	(NE-EDR sh2/Blue Indian) F_5_ + (NE-EDR sh2/Bloody Butcher) F_5_
H9	CS3+CS4	(P39 su1/Blue Indian) F_5_ + (IA5125 su1/Bloody Butcher) F_5_
H10	CS3+CS6	(P39 su1/Blue Indian) F_5_ + ((Double Red Sweet corn//(NE-EDR su1/Blue Indian) F_2_) F_5_
H11	CS3+CS7	(P39 su1/Blue Indian) F_5_ + ((NE-EDR sh2/Blue Indian) F2//Double Red Sweet corn)) F_5_
H12	CS3+CS8	(P39 su1/Blue Indian) F_5_ + (NE-EDR sh2/Bloody Butcher) F_5_
H13	CS3+CS9	(P39 su1/Blue Indian) F_5_ + (NE-EDR su1/Bloody Butcher) F_5_
H14	CS4+CS5	(IA5125 su1/Bloody Butcher) F_5_ + ((NE-EDR *sh2*/BI)//Seneca Red Stalker) F_5_
H15	CS4+CS6	(IA5125 su1/Bloody Butcher) F_5_ + ((Double Red Sweet corn//(NE-EDR su1/Blue Indian) F_2_) F_5_
H16	CS4+CS8	(IA5125 su1/Bloody Butcher) F_5_ + (NE-EDR sh2/Bloody Butcher) F_5_
H17	CS4+CS9	(IA5125 su1/Bloody Butcher) F_5_ + (NE-EDR su1/Bloody Butcher) F_5_
H18	CS5+CS9	((NE-EDR *sh2*/BI)//Seneca Red Stalker) F_5_ +
H19	CS6+CS9	(Double Red Sweet corn//(NE-EDR su1/Blue Indian) F_2_) F_5_ + (NE-EDR su1/Bloody Butcher) F_5_
H20	CS8+CS9	(NE-EDR sh2/Bloody Butcher) F_5_ + (NE-EDR su1/Bloody Butcher) F_5_

20 total CS hybrids were developed in the winter of 2024. For simplicity, reference numbers H1-H20 were used for identification of hybrids in this analysis.

### Harvesting, processing, and storage of eating stage sweet corn

For evaluation and selection analyses, sweet corn ears were harvested at 20 DAP. Four to six biological replicates of each genotype were hand-picked and transported in a cooler to the laboratory for long-term storage. Before freezing, replicates were husked, arranged with a label showing their genotype and pollination date, digitally imaged, and placed in liquid nitrogen. Each frozen ear was transferred to individual labelled resealable bags and larger bags for organization and then stored at -80° Celsius.

### Carotenoids, flavonoids and phenolic acids content measurements

#### Chemicals and standards

Acetone (A9491), methanol (A4564), acetonitrile (A9554), ethyl acetate (E1954) and acetic acid (A38212) were obtained from Fisher Scientific (Pittsburgh, Pennsylvania, USA). Analytical standards obtained from Sigma-Aldrich (St. Louis, MO, USA) include alpha-cryptoxanthin (69553), beta-cryptoxanthin (C6368), neoxanthin (72994), alpha-carotene (50887), beta-carotene (PHR1239), caffeic acid (C0625), catechin (PHR1963), chlorogenic acid (C3878), delphinidin (43725), epicatechin (E4018), ferulic acid (128708), kaempferol (60010), p-coumaric acid (C9008), phloretin (P7912), protocatechuic acid (PHL89766), quercetin (Q4951), quercetin-3-glucoside (17793), resveratrol (R5010), rutin (PHL89270), and vanillic acid (94770). Apigenin (AAL15041MB), luteolin (AAL14186MC), naringenin (AAL0983404), cinnamic acid (AC158570050), daidzein (AC328230250), gallic acid (AC410860050), genistein (AC328270250), and syringic acid (AC132890100) were purchased from Fisher Scientific (Pittsburgh, Pennsylvania, USA). Lutein (10010811) and zeaxanthin (10009992) were obtained from Cayman Chemicals (Ann Arbor, Michigan, USA), and violaxanthin (PPS-VIOL) and antheraxanthin (PPS-ANTH) from DHI Lab Products (Denmark).

#### Extraction and determination of carotenoids

Carotenoid compounds, alpha-carotene, beta-carotene, antheraxanthin, lutein, violaxanthin, neoxanthin, zeaxanthin, alpha cryptoxanthin, and beta-cryptoxanthin were profiled and analyzed in fresh colored sweet corn inbreds. A minimum of eight frozen kernels were picked from three biological replicate ears and were lyophilized from frozen for 48 hours. Dry kernels were then ground to a fine flour in liquid nitrogen. Samples were transferred to a 2 mL centrifuge tube and were stored at -20 °C. The carotenoids were extracted and quantified using modified protocol described by Haupt and Glowacka, 2024. In detail, 40 ± 0.5 mg flour samples were weighed, and a chilled stainless steel bead was added to the samples and extraction blank tube. Samples were homogenized using a tissue lyser for three minutes at 20 Hz. Samples were then placed on dry ice while adding 500 μL of acetone and immediately capped the tubes to avoid acetone evaporation. Tubes were then transferred to the chilled tissue lyser rack, and the contents were homogenized for 10 minutes at 20 Hz. Immediately, homogenized contents were centrifuged at 4 °C for 5 minutes at 16,000 g. Tubes were placed on a cool rack or dry ice while the supernatant were transferred to a new 2 mL tubes. Extraction was repeated on the pellet and the supernatants were combined. Again, the total supernatant was centrifuged at 4 °C for 20 minutes at 16,000 g and then transferred to an HPLC vial. The sample contents in the HPLC vial were evaporated under a clean stream of nitrogen gas using the Microvap Microplate Evaporator. Samples were resuspended in acetone (resuspension ratio of 20 mg starting material to 100 mL acetone) and were mixed thoroughly. Thirty microliters of the samples were then transferred into clear vials and carotenoids separated using liquid chromatography and detected using Diode Array Detector (DAD) at 440 nm.

Quantification was performed using carotenoid standards prepared at multiple concentrations to generate external calibration curves. Linearity was evaluated across the calibration range, and concentrations of individual carotenoid compounds were determined from the resulting linear regression models.

#### Extraction and determination of flavonoids and phenolic acids

The flavonoid compounds associated with kernel color pigmentation were profiled and analyzed. Fifty mg flour samples were weighed and homogenized (as described above in the carotenoid extraction section). The flavonoids and phenolic acids were extracted and quantified using modified protocol described by Barboza Bispo et al., 2024. A volume of 900 μL of 100% methanol was added to the samples and a blank tube followed by 5 μL of 100 μM synthetic strigolactone GR24 as internal standard. Samples were re-homogenized in the tissue lyser for 15 minutes at 10 Hz. Samples were then centrifuged at 4 °C for 10 minutes at 16,000 g and supernatants transferred to 2 mL tubes and placed on ice. The solvent extraction step was repeated by adding 900 μL of methanol to the remaining pellet, then centrifuged for 10 minutes at 16,000 g. The supernatant was combined to the first supernatant. The samples were dried down in a Speedvac. Dried samples were stored at -80 °C until ready for resuspension. Samples were resuspended in 100 μL of 30% methanol for 30 minutes on a platform shaker. Resuspended samples were then centrifuged for 20 minutes at 16,000g and 30 μL of each sample transferred to an HPLC vial to be analyzed using LC-MS/MS (liquid chromatography-tandem mass spectrometry). Flavonoids and phenolic acids were separated using an Agilent ZORBAX Eclipse Plus C18 column and were detected using multiple reaction monitoring on a QTRAP 6500+ mass spectrometer as previously described (*Molecules* 2021, *26*(21), 6496; https://doi.org/10.3390/molecules26216496). The compounds detected included apigenin, caffeic acid, catechin, cinnamic acid, chlorogenic acid, daidzein, delphinidin, epicatechin, ferulic acid, gallic acid, genistein, kaempferol, p-coumaric acid, luteolin, naringenin, phloretin, protocatechuic acid, quercetin, quercetin-3-glucoside, resveratrol, rutin, syringic acid, and vanillic acid. An external standard curve was prepared using multiple standard sample of different concentrations of unlabeled compounds and fixed concentration of the internal standard. Analyst 1.6.3 software were used to analyze the data.

### Determination of sugar and starch content of colored sweet corn inbreds and hybrids

The sugar contents in colored sweet corn inbreds and hybrids were determined using the Megazyme Sucrose/D-Glucose Assay Protocol (CAT NO. K-SUCGL) and Total Starch Assay Protocol (REF K-TSTA-100A; 700004351). A minimum of eight frozen kernels were picked from four to six biological replicate ears and were lyophilized from frozen for 48 hours. Dry kernels were then ground to a fine flour in liquid nitrogen. Samples were transferred to a 2 mL centrifuge tube and were stored at -20 °C. To determine the sucrose content, 50 mg samples were used and were diluted to 0.5 mg/mL using deionized water. An aliquot of 0.2 mL sample was added to two duplicate sets of 16 x 10 mm glass test tubes. A volume of 0.2 mL of acetate buffer (included in the kit) and of b-fructosidase were added to duplicate tubes. Samples, reagent blanks, and controls were incubated at 50 °C for 20 minutes. A volume of 3.0 mL of Glucose Oxidase/Peroxidase (GOPOD) reagent buffer was added to all tubes and tubes were incubated again at 50 °C for 20 minutes. Immediately, absorbances of samples and controls were measured at 510 nm against the reagent blank using a MegaQuant™ Wave Spectrophotometer. To measure the total starch content, 100 mg of sample were washed with 80% v/v aqueous ethanol and dimethyl sulphoxide to remove free D-Glucose or maltodextrins and resistant starch respectively. Diluted thermostable α-amylase (1 mL of α-amylase in 30 mL of MOPS) was then added to samples to extract D-Glucose, followed by incubation in boiling water for six minutes while thoroughly mixing every two minutes. Samples were placed in a 50° C bath and 4 mL of sodium acetate buffer [(200mM, pH 4.5) plus calcium chloride (5 mM)] was added, followed by 0.1 mL of Amyloglucosidase. Then, samples were incubated for 30 minutes. After, sample contents were transferred to a 100 mL volumetric flask and adjusted to volume with. acetate buffer. 0.1 mL of final sample contents were added to duplicate tubes and 3 mL of GOPOD reagent were added and incubated at 50° C for 20 minutes. The reaction of the sample solutions with the GOPOD reagent produces quinoneimine dye whose absorbance values were measured using Megazyme^®^ Wave Spectrophotometer. The absorbance values were used to calculate the sucrose and total starch content using the Megazyme’s Mega-calc tool.

### Preliminary sensory evaluation of color, sweetness and texture of colored sweet corn inbreds and hybrids

Kernel color, sweetness, and texture were evaluated in fresh colored sweet corn at 20 DAP to examine how well the new colored sweet corn inbreds and hybrids maintained the primary sweet corn kernel palatability given the additional non-sweet corn backgrounds. Three to five colored sweet corn ears were picked and were immediately tasted raw by at least three testers. At least three separate ears were tested to account for plant to plant variation. Then, a consensus score for color (1 being white/yellow, 5 being any color besides white/yellow), taste (1 being bland, 5 being sweet) and texture (1 being tough, 5 being tender) were assigned. Moreover, plants were visually evaluated for their physical phenotypes such as height, pollen quality, ear size and weight, and early/late pollination period. These qualities were used in selection of superior colored sweet corn lines at each generation and later used in hybrid production.

### Statistical analysis

Sucrose and total starch data were presented as means ± SEM. A one-way analysis of variance (ANOVA) was performed to determine statistical differences between parental sweet corn, colored sweet corn inbreds and hybrids followed by Tukey’s Honestly Significant Difference (HSD) multiple comparison test. Statistical significance was shown as asterisks: *p < 0.05; **p < 0.01; ***p < 0.001, and ns: not significant.

## Results

### Production of colored sweet corn

**Figure 2:**
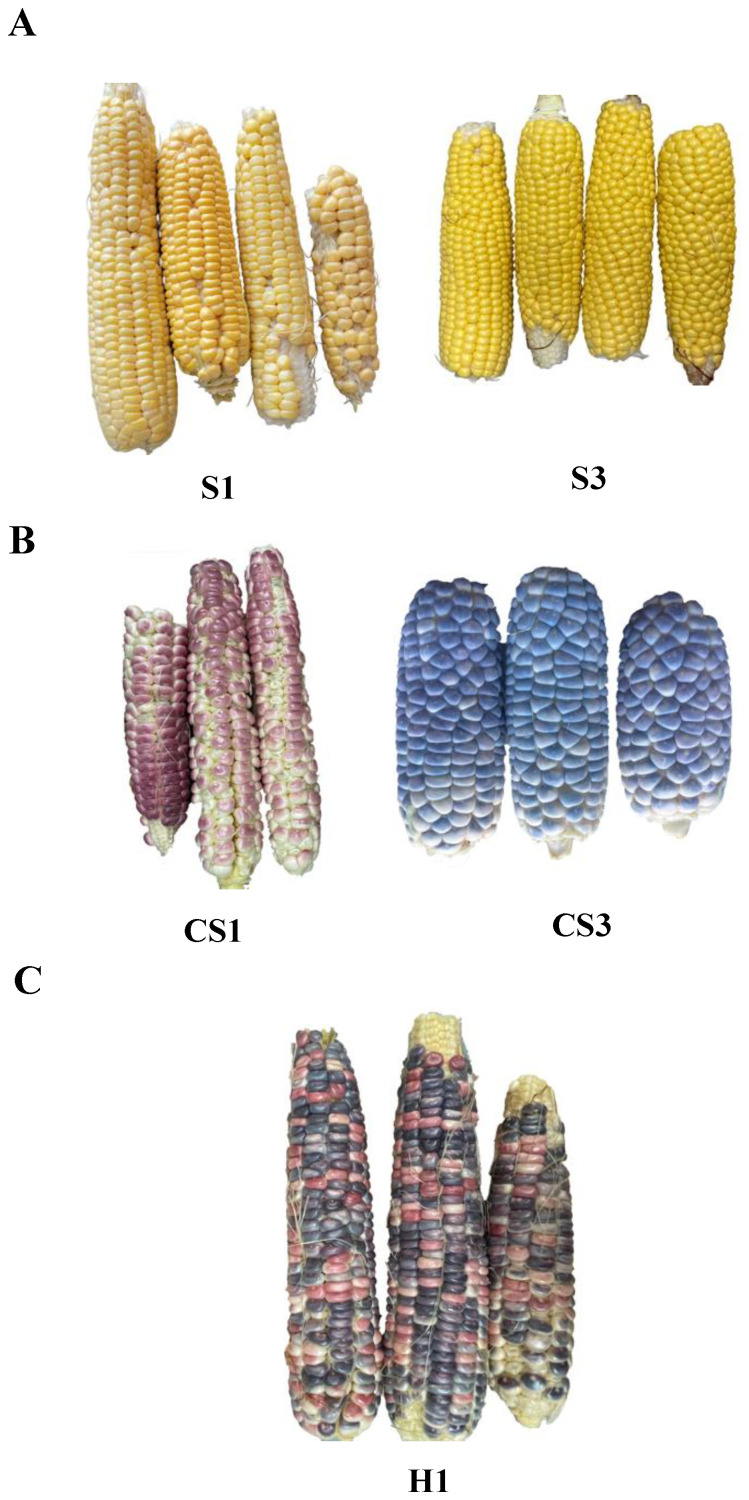
Freshly harvested sweet corns at 20 DAP. **(A)** Parental sweet corns (S1: NE-EDR su1 and S3: P39 *su1*). **(B)** Colored sweet corn inbreds (CS1: NE-EDR *su1* x Blue Indian and CS3: P39-*su1* x Blue Indian). **(C)** Colored sweet corn hybrid (H1: CS1+CS3).

Colored maize and sweet corn parent lines are shown in **Supplementary Table 1** and the breeding scheme is shown in **Figure 1**. It took five generations of selfing to transform the yellow parental sweet corns (**Figure 2A**; **Supplementary Figure 1**) into colored sweet corns (**Figure 2**; **Supplementary Figures 1**, **S2**). Nine CS inbreds and 20 CS hybrids were produced. Five CS inbreds (CS1, CS3, CS4, CS6, and CS9) carried the *su1* sweet corn mutation while four CS inbreds (CS2, CS5, CS7, and CS8) carried *sh2* sweet corn mutation (**Table 1**). These inbreds produced 20 CS hybrids where eight CS hybrids (H1, H2, H4, H9, H10, H13, H15, and H19) had su1 parental inbreds, three CS hybrids (H7, H8, and H17) had sh2 parental inbreds, and nine CS hybrids (H3, H5, H6, H11, H12, H14, H16, H18, and H20) had one inbreds carrying *su1* and the other carrying *sh2* allele (**Table 2**). The early generation lines displayed weak color formation at sweet corn prime eating stage and the vivid color pigments formed especially at maturity. Due to lack of visible color formation in earlier generations, the selection of color phenotypes was performed on mature lines. However, the kernel pigments got more vivid in later generations allowing the selection of colored sweet corn at its prime eating stage. The continuous selection of specific color phenotype often led to the fixation of a single color phenotype in CS at the F_5_ inbred phase (**Figure 2B**; **Supplementary Figure 1**). Purple, red, blue and orange were the most prevalent colors formed in the final inbred phase. The CS inbreds were classified into bright-colored and dark-colored kernel pigment classes. CS2, CS4, and CS8 displayed orange and yellow orange like pigments whereas CS1, CS3, CS5, CS6, CS7, and CS9 displayed blue, purple, and red pigments. Only CS lines that displayed visible color formation at 20 DAP were selected to generate parental inbred lines used in the production of the CS hybrids. The CS hybrids conserved the color phenotypes of the parental inbreds (**Figure 2C**; **Supplementary Figure 2**). The uniform color formation in the resultant colored sweet corn inbreds and hybrids provided the proof-of-concept that conventional breeding approach may be used to confer kernel pigments in eating stage sweet corn from non-sweet corn varieties.

### Preliminary evaluation of kernel palatability shows comparable kernel sweetness and texture among CS inbred, hybrid, and parental sweet corn lines

The CS lines were evaluated for kernel color visibility, sweetness, and texture to assess the effects of crossing to colored non-sweet corn lines. This evaluation was used to identify the best lines at each generation and the selected colored sweet corn lines displayed satisfactory kernel color intensity, sweetness, and texture at its eating stage. An arbitrary scoring scale of 1 to 5 was employed to test at least three fresh colored sweet corns and parental sweet corns. A threshold score of 3 was the bare minimum requirement in selecting good lines. The genotypes that showed lower scores than 3 were discarded and were not advanced to the next generation (**Supplementary Table 2**). In the earlier generations, the majority of the lines with popcorn and flint corn backgrounds displayed diverse color formations, but they also had bland taste and tougher texture. This led to poor sweet corn sugary quality traits, and they were discarded. Only a few non-sweet corn lines such as Blue Indian, Bloody Butcher, Seneca Red Stalker displayed consistent satisfactory scores throughout the generations to the final F_5_ inbred phase. The selection of sweet and tender colored sweet corn lines led to the production of nine CS inbreds. The kernel eating qualities in CS inbreds were comparable to the parental sweet corn in terms of sweetness and tenderness (**Table 3**). A controlled crossing among these CS inbreds generated 20 F_1_ CS hybrids and the evaluation of the F_2_ lines derived from the F_1_ hybrids confirmed the consistency of kernel color, sweetness, and tenderness derived from the parental inbreds. Eight *su1* CS hybrids and three *sh2* CS hybrids showed high scores equivalent and, in some cases, better than the parental CS inbred lines (**Table 4**). Some hybrids were assigned a score of 6 to highlight their exceptional performance. Furthermore, nine CS hybrids, which were produced from parental inbreds where one carried *su1* and the other carried *sh2*, showed kernel palatability scores comparable to parental inbreds with H5, H6, and H18 showing tougher kernel texture (**Supplementary Table 3**). The F_1_ hybrids were heterozygous Su1su1Sh2sh2 and the F_2_ lines segregated 9/16 wild type and 7/16 sweet corn genotypes. The evaluation of kernel palatability clearly included both wild type and sweet corn kernels. Though less ideal for commercial setting, these hybrids may be suited for further production of double mutant sweet corn lines. Overall, the sensory test of the resultant colored sweet corn indicates that, with careful selection for sweet corn qualities, it is possible to breed diverse color into sweet corn from some non-sweet corn colored lines and still conserve traditional sweet corn sweet and tender phenotypes.

**Table 3:** Palatability scores in parental and colored sweet corn inbreds.

Lines	Color		Sweetness		Texture	
	Parental sweet corn	Colored sweet corn	Parental sweet corn	Colored sweet corn	Parental sweet corn	Colored sweet corn
S1 & CS1	1	5	4	5	4	5
S2 & CS2	1	5	5	5	5	5
S3 & CS3	1	5	4	4	4	5
S4 & CS4	1	4	5	3	4	5
S2 & CS5	1	4	5	5	5	5
S1 & CS6	1	5	4	5	4	5
S2 & CS7	1	5	5	4	5	5
S2 & CS8	1	3	5	5	5	5
S1 & CS9	1	5	4	4	4	4

Color, sweetness, and texture scores for parental sweet corn and colored sweet corn inbreds evaluated during the Summer 2023. Freshly harvested ears (20 DAP) (n=3) were scored for color (1, yellow/white; 5, purple, blue, red…) sweetness (1, bland; 5, sweet) and texture (1, tough; 5, tender).

**Table 4:** Palatability scores in F_2_ lines derived from F_1_ CS hybrids.

Hybrids	Color score (1–5)	Sweetness score (1–5)	Texture score (1–5)	Phenotypic descriptions
H1	6	6	5	Mixed colors, very sweet
H2	6	6	6	Tall plants, large ears, early pollination
H3	4	5	3	Large plants and ears, mixed colors, but somewhat tough and mealy
H4	5	5	5	Mixed colors, sweet, tender
H5	6	5	3	Bronze color, sweet, tough
H6	6	6	3	Big ears but tough texture
H7	5	5	5	Tall plants, sweet and tender
H8	5	5	6	Tall plants, sweet, crispy
H9	5	2	4	Nice tan color
H10	6	5	6	Beautiful & early color, deep red
H11	5	4	4	–
H12	5	5	5	–
H13	5	6	5	Mixed color: yellow, pink and purple. Good flavor, tall plants and big ears
H14	6	5	5	Bronze/red color, rainbow
H15	6	4	5	Early, tall, fat eats, bronze red.
H16	5	5	5	–
H17	5	5	5	–
H18	5	5	3	Very sweet but tough
H19	6	6	6	Winner. Pink/red color
H20	5	6	4	–

Color, sweetness, and texture scores for 11 Colored sweet corn hybrids (su1 and sh2 types) evaluated during Summer 2024. Freshly harvested ears (20 DAP) (n=3) were scored for color (1, yellow/white, 5, purple, blue, red…) sweetness (1, bland, 5, sweet) and texture (1, tough, 5, tender).

### Confirmation of sucrose content in colored sweet corn

**Figure 3:**
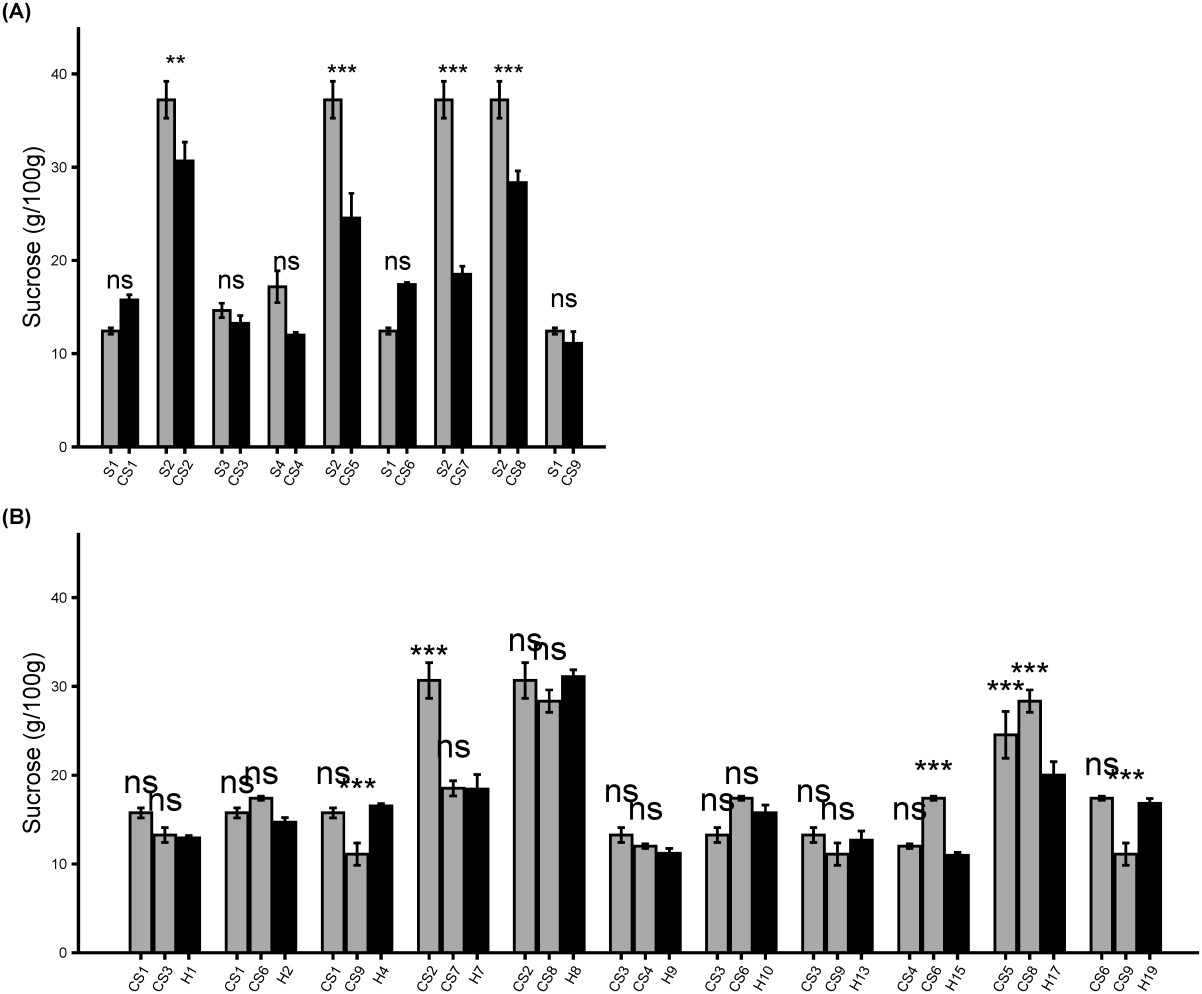
Sucrose biochemical analysis. **(A)** Sucrose content (g/100g) in parental sweet corn parents and derived colored sweet corn inbreds. **(B)** Sucrose content (g/100g) in colored sweet corn inbreds and derived hybrids. Data represent means ± SE (n = 4). Statistical difference between parental sweet corn, colored sweet corn inbreds and hybrids was determined using one-way ANOVA followed by Tukey’s HSD test. The asterisks show significant differences: *p < 0.05; **p < 0.01; ***p < 0.001, and ns, not significant.

We investigated how the introgression of color pigments into the *su1* and *sh2* sweet corn inbreds using colored maize varieties of non-sweet corn background affected the sugar content of the new colored sweet corn inbreds and hybrids. The sucrose content of the lyophilized fresh colored sweet corn inbreds and hybrids were measured and analyzed (**Figure 3**). Five colored sweet corn inbred introgressions did not significantly differ from the parental sweet corn (p> 0.05). In contrast, the sucrose content was significantly lower in the four *sh2* colored sweet corn inbreds compared to the parental sweet corn line (p<0.05). Even though the sucrose content were lower in *sh2* inbred lines, it was still higher than the sucrose content of the *su1* inbred lines (**Figure 3A**). The differences in sucrose content between *su1* and *sh2* inbred lines may be attributed to the differences in the sucrose content of the parental sweet corn lines where *sh2* sweet corn parent has higher sucrose content than the *su1* sweet corn parent. Within hybrids, there was a varying degree of sucrose content among CS hybrids depending on the sweet corn *su1* and *sh2* mutations present in the parental inbreds. Eleven CS hybrids, where both parental inbreds shared one sweet corn mutation (eight *su1* and three *sh2* types), had sucrose content relatively equivalent to both of parental inbreds except for H4, H7, H15, H17, and H19. H4 and H19 showed significantly higher sucrose than one of the parental inbreds whereas H7 and H15 had significantly lower sucrose than one of the parental inbreds (*p* < 0.05) (**Figure 3B**). Additionally, for the nine CS hybrids that segregated from wild type and sweet corn, results showed that they had sucrose content leaning closely to that of the *su1* inbred than the *sh2* inbred (**Supplementary Figure 3**). Sucrose was measured from F_2_ ears and samples most certainly included wild type kernels since sweet corn cannot be differentiated from wild type at this stage. This might have lowered the sucrose content of the hybrids.

### Confirmation of total starch content in colored sweet corn

**Figure 4:**
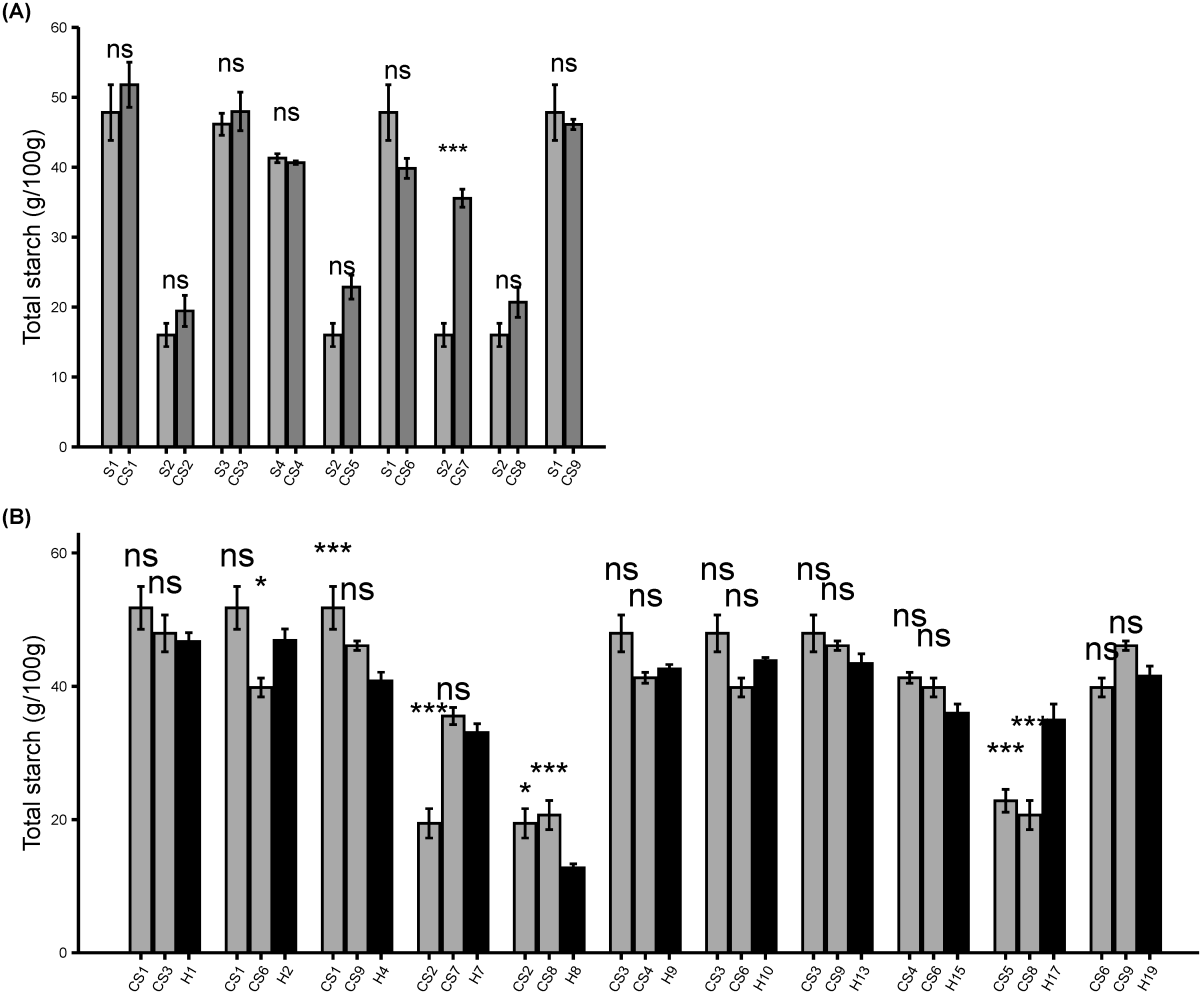
Total starch Biochemical Analysis. **(A)** Total starch content (g/100g) in parental sweet corn and derived colored sweet corn inbreds. **(B)** Total starch content (g/100g) in colored sweet corn inbreds and derived hybrids. Data represent means ± SE (n = 4). Statistical difference between parental sweet corn, colored sweet corn inbreds and hybrids was determined using one-way ANOVA followed by Tukey’s HSD test. The asterisks show significant differences: *p < 0.05; **p < 0.01; ***p < 0.001, and ns, not significant.

Due to the high starch content of the colored maize lines used in the initial crosses, the total starch test was performed to measure the effects of crossing lines with high starch background into sweet corn. The results showed that both *su1*- and *sh2-* derived CS inbreds had total starch content relatively equivalent to the parental sweet corn except CS7 inbreds which had significantly higher starch than the respective *sh2* sweet corn parent (*p* < 0.05) (**Figure 4A**). The starch content was still lower in *sh2* inbreds than the *su1* inbreds. Similar to the inbreds, the CS hybrids had lower starch in the *sh2* than the *su1* lines (**Figure 4B**). The starch in s*u1* CS hybrids remained consistently similar to parental inbreds except H2 and H4 which had significantly higher and lower starch than one of the parental inbreds, respectively (*p* < 0.05). In contrast, the *sh2* CS hybrids H7 and H17 had significantly higher starch than one or both parental inbreds except H8 which had significantly lower starch than both parental sweet corn (*p* < 0.05). For CS hybrids crosses where both *su1* and *sh2* were present, the starch content tended to resemble the levels of *su1* parental inbred (**Supplementary Figure 4**). Similar to sucrose, samples were a mixture of wild type and sweet corn kernels; thus, the increased starch content in the segregating CS hybrids were most likely attributable to the highly starch content of the wild type.

### Carotenoid contents in colored sweet corn inbreds

**Figure 5:**
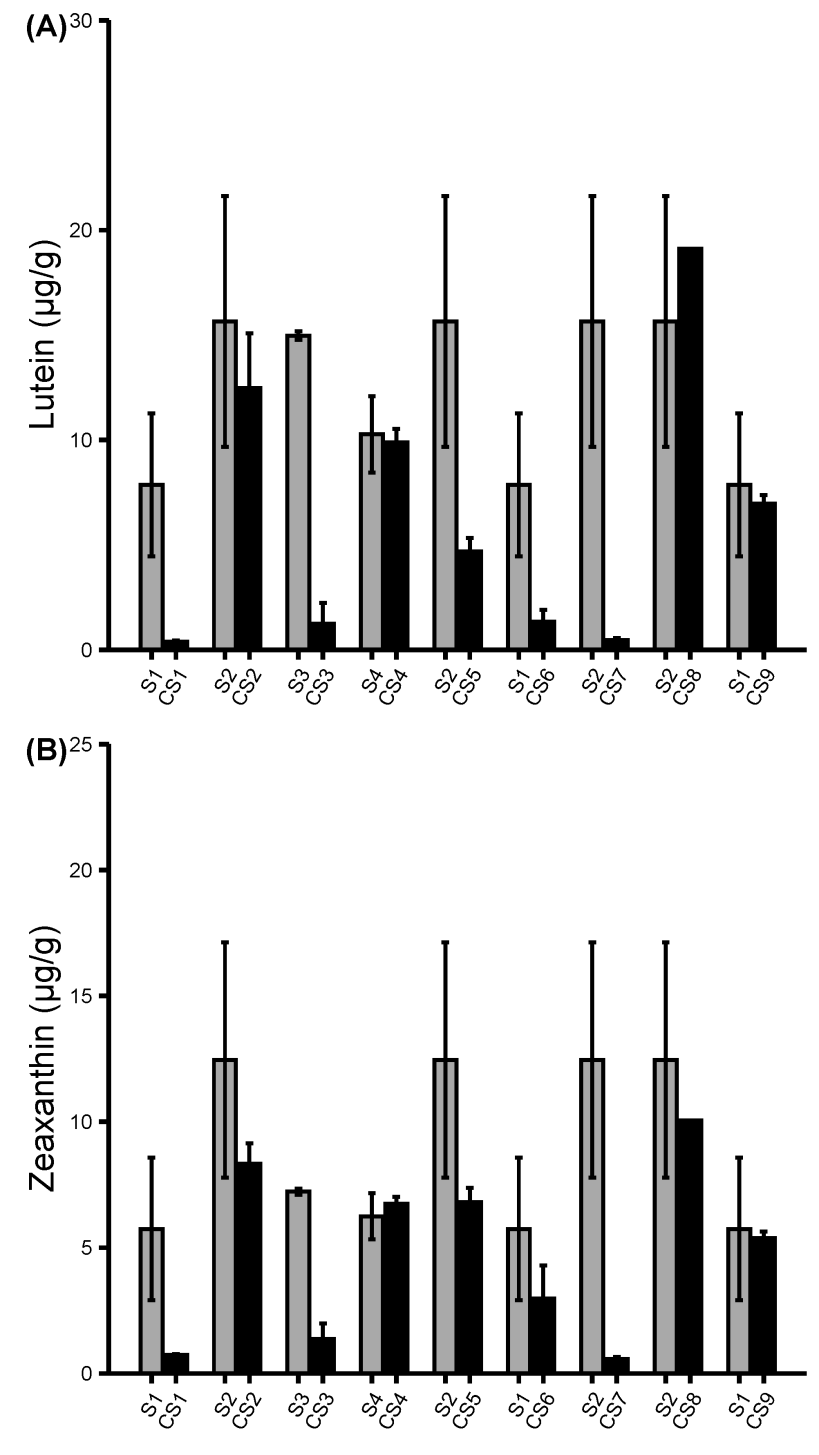
Profiling of carotenoids. **(A, B)** concentrations of the carotenoid compounds in parental sweet corn and colored sweet corn inbreds. Data represent means ± SE (n = 3). Additional carotenoid compounds were shown in **Supplementary Figure 3**.

To determine whether the introgression of kernel color in sweet corn changed its carotenoid profile, HPLC was used to profile and quantify the concentrations of the carotenoid compounds in colored sweet corn. Nine carotenoid compounds were detected in the CS inbreds (**Figure 5**; **Supplementary Figure 5**). The carotenoids detection differed substantially according to the genotype’s color types. β-carotene, antheraxanthin, lutein, violaxanthin, neoxanthin, and zeaxanthin were mostly detected in all the nine CS inbreds while alpha-carotene, α-cryptoxanthin, and β-cryptoxanthin were most predominant in the genotypes with the orange and orange-red colored kernels. CS2, CS4, and CS8 inbreds comprised of colors ranging from yellowish orange to yellowish red and purple, suggesting that there may be carotenoid pigments accumulated in the kernels. The results showed that the carotenoid pigments increased mostly in the CS2 and CS4 inbreds. Alpha carotenes in CS2 and CS4 increased by 22.64% and 5.35% respectively while the β-carotenes increased by 62.75% and 14.97% respectively. In addition, the xanthophylls, including beta cryptoxanthin increased in CS2 and CS4 by 32.38% and 27.24% respectively. In contrast, the pigment concentrations in the dark-colored CS inbreds like CS1, CS3, CS6, CS7, and CS9 were substantially reduced or not present compared with their respective sweet corn parents. Generally, the yellow, orange, and red-orange colored kernels exhibited substantially higher carotenoid concentrations compared with the blue, purple, and red colored kernels. Overall, the CS lines of similar kernel color pigments exhibited different concentrations of the carotenoid compounds indicating possible varying levels of pigments accumulation among the CS lines.

### Flavonoids and phenolic acids content in colored sweet corn inbreds

**Figure 6:**
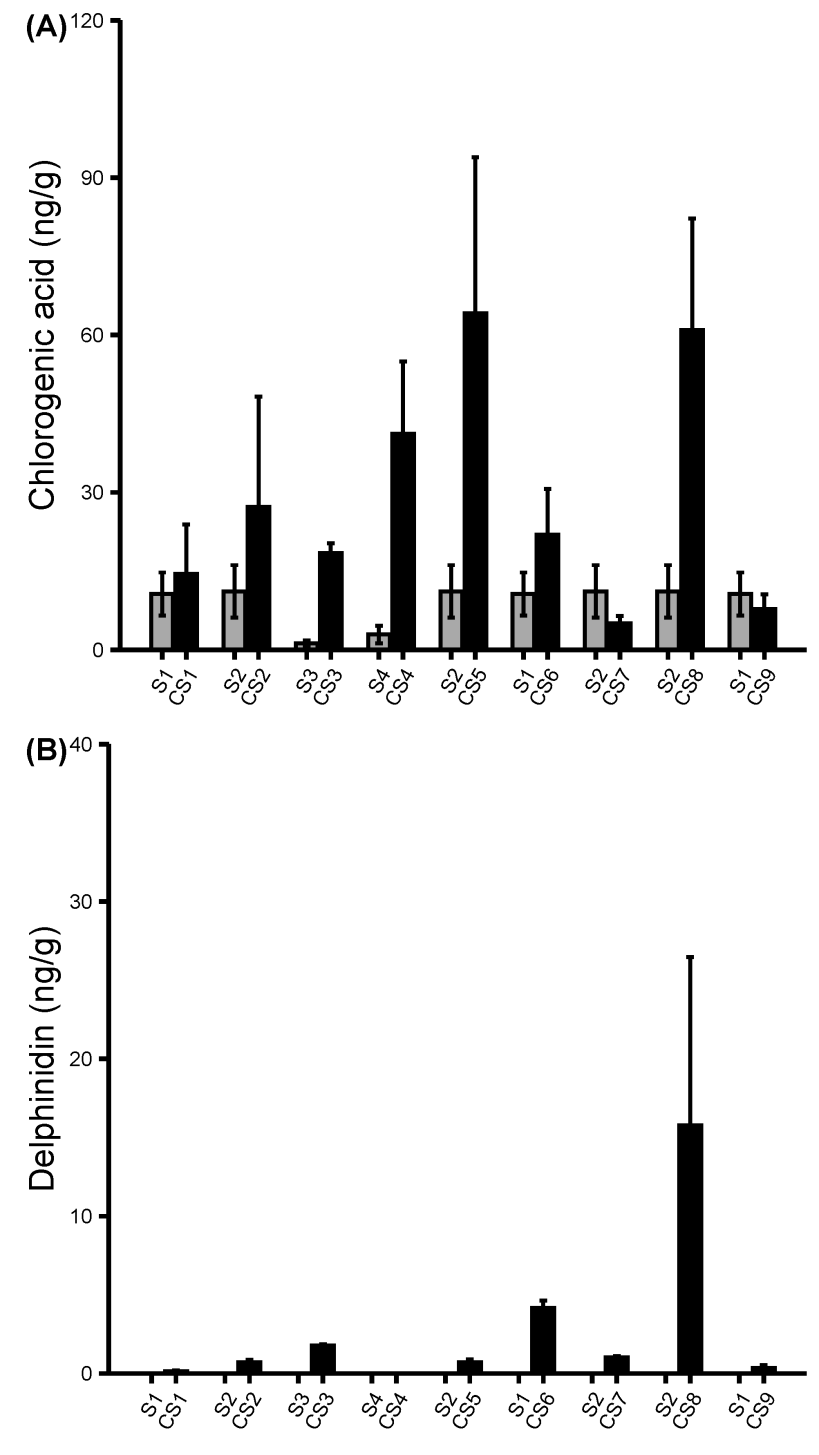
Profiling of flavonoids. **(A, B)** concentrations of the flavonoid compounds in parental sweet corns and colored sweet corn inbreds. Data represent means ± SE (n = 3). Additional flavonoid compounds were shown in **Supplementary Figures 4**, **S5**.

**Figure 7:**
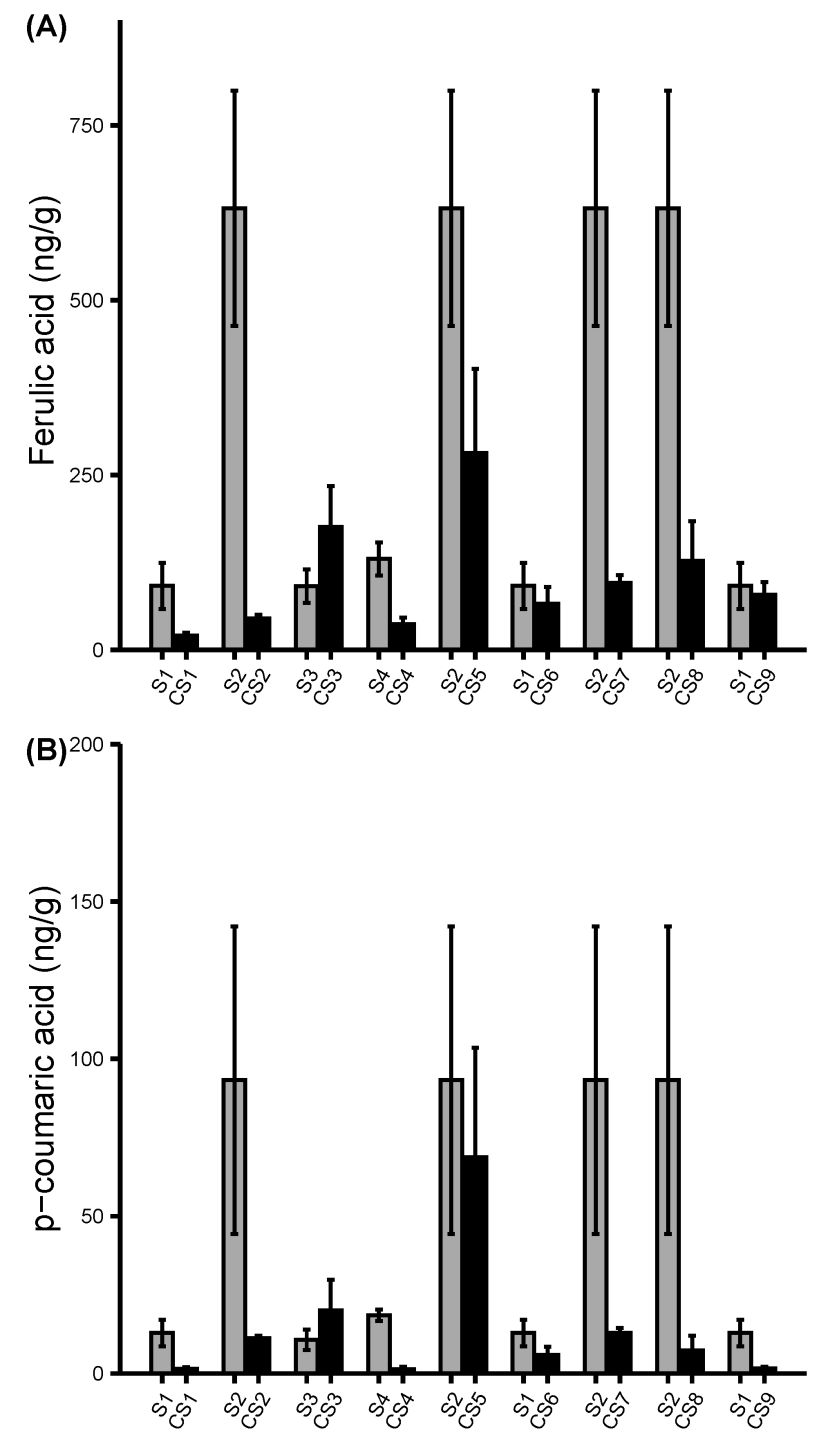
Profiling of phenolic acids. **(A, B)** concentrations of the phenolic acids in parental sweet corns and colored sweet corn inbreds. Data represent means ± SE (n = 3). Additional phenolic acid compounds were shown in **Supplementary Figure 6**.

The predominant kernel colors observed in six out of the nine final colored sweet corn lines were purple, blue, and red. These colors pigments predict the presence of the flavonoid compounds. Therefore, the pigment compounds were extracted, and their concentrations were measured using targeted LC-MS/MS to assess the effects of introducing color pigments on sweet corn’s flavonoids content profile. A total of 23 flavonoids and phenolic acids were profiled from the colored sweet corn inbreds. The concentrations of nine flavonoid compounds were consistently higher in colored sweet corn lines compared with the parental sweet corn (**Figure 6**; **Supplementary Figures 6**, **S7**). Unlike the carotenoids which were abundantly detectable in the parental sweet corn, only few flavonoids like chlorogenic acid and luteolin were detectable at low levels but mostly the flavonoids were not found at all in parental sweet corn. The dark-colored pigments led to increased concentrations of the flavonoid compounds such as delphinidin, naringenin, luteolin, quercetin, resveratrol, rutin, and apigenin in most of the CS inbreds. The effects were stronger in CS inbreds such as CS1, CS3, CS5, and CS6 where purple, blue, and red kernel colors were predominant. However, some CS inbreds were observed to have little to no detectability for certain flavonoid compounds (**Supplementary Figure 7**). For instance, there were elevated concentrations of apigenin, genistein, and kaempferol compounds in CS6 genotype but these compounds were not detected in other genotypes of similar color phenotype as CS6. Besides the flavonoids, the phenolic acids were measured in CS inbreds (**Figure 7**; **Supplementary Figure 8**). Results showed that parental sweet corns had higher concentrations of phenolic acids than the colored sweet corn inbreds, except CS3 inbred which was the only inbred with blue color. Overall, the introgression of color pigments in sweet corn increased the concentration of the flavonoid compounds and decreased the concentrations of the phenolic acids. Additionally, the concentration of the flavonoids substantially differed among the colored sweet corn genotypes of the same pigments suggesting that visible color was not the only predictor of the total flavonoid compound concentration.

### Evaluation of ear agronomics in the F_2_ lines derived from the F_1_ colored sweet corn hybrids

Along with assessing the kernel palatability qualities of the fresh 20 DAP CS hybrids, the ear weight and length of F_2_ lines derived from the F_1_ CS hybrids were measured to assess agronomic yield-related traits. The average ear weight ranged from 154.8 ± 46.6 to 252.81 ± 39.3 g (**Table 5**) while the average ear length ranged from 128 ± 15 to 198 ± 10 mm (**Table 6**). Tukey’s HSD multiple comparisons among the CS hybrids ear weight and length showed no significant differences across the CS hybrids except H13 and H15 (p<0.05) (**Supplementary Tables 4**, **S5**). The ear weight was proportional with the ear length in most hybrids, indicating that longer ears mostly weighed more. In some cases, being long did not automatically translate into heavier ears. Hybrid H4 was 51 mm longer but 80.99 g lighter than H15. H4 was scrawny and tall while H15 was robust and short. Though there was less variation in the average ear weight and length among the CS hybrids, high variation in ear weight and length was observed within the same hybrid lines. For instance, the biological replicates weight of H3, H8, and H19 had weights of 55.8g, 62.3g, and 54.5g, respectively resulting in high standard deviations. And the biological replicates lengths of H3, H12, H20 had lengths of 46 mm, 36 mm, and 41 mm, respectively resulting in high standard deviations. These hybrids were planted in the same field with same conditions; thus, the variation in ear weights and lengths pertains to the hybrid itself. F_1_s hybrids (developed bidirectionally) were bulked to produce the F_2_ lines on which the seed agronomics were performed. Assuming homogeneous field conditions, the plant-to-plant variations can be attributed to the differences of the hybrid seeds used. On the average, the CS hybrids performed relatively similar in terms of ear agronomics. Generally, short ear hybrids did not mean poor overall ear agronomic performance. Both ear length and weight contributed to the overall performance of the colored sweet corn hybrids.

**Table 5:** Ear weight of 20 DAP F_2_ lines derived from F_1_ CS hybrids.

Weight (g)								
Hybrids	Ear 1	Ear 2	Ear 3	Ear 4	Ear 5	Ear 6	Mean	*sd*
H1	207.7	197.1	155.3	124.6	149.5	194.2	171.4	33.0
H2	173.8	189.5	251.8	152.8	171.8	196.6	189.4	34.2
H3	220.5	263.4	150.6	147.9	135.1	–	183.5	55.8
H4	180.0	167.4	205.1	217.0	145.0	104.4	169.8	41.2
H5	230.2	233.5	246.8	218.9	228.1	236.8	232.4	9.2
H6	174.8	145.8	194.8	194.7	212.2	195.1	186.2	23.1
H7	208.8	144.1	140.9	231.2	185.7	111.7	170.4	45.6
H8	249.3	154.1	285.9	280.8	298.0	170.9	239.8	62.3
H9	235.9	283.5	265.5	214.8	231.8	239.4	245.1	24.9
H10	219.0	187.8	237.8	233.3	201.7	177.5	209.5	24.5
H11	206.1	224.4	267.3	196.4	207.1	265.5	227.8	31.2
H12	202.4	178.7	184.5	303.9	244.9	238.7	225.5	47.1
H13	198.9	217.7	259.6	182.0	256.2	236.1	225.1	31.2
H14	228.4	210.5	234.9	221.5	257.6	271.5	237.4	22.9
H15	249.8	245.8	228.2	234.4	330.9	227.9	252.8	39.3
H16	292.4	217.4	191.0	213.2	249.9	222.3	231.0	35.5
H17	245.5	235.7	235.7	–	–	–	240.6	6.89
H18	129.7	205.5	232.2	179.1	162.8	191.2	183.4	35.4
H19	261.7	263.5	294.45	183.3	1678.0	177.7	224.7	54.5
H20	132.1	205.1	220.7	142.0	110.1	118.9	154.8	46.6

Data represent means ± SE (n = 6). Statistical difference in ear weight of colored sweet corn hybrids was shown in **Supplementary Table 4**.

**Table 6:** Ear length of 20 DAP F_2_ lines derived from F_1_ CS hybrids.

Hybrids length (mm)								
Hybrids	Ear 1	Ear 2	Ear 3	Ear 4	Ear 5	Ear 6	Mean	*sd*
H1	200	205	180	150	160	160	176	23
H2	160	190	180	160	170	170	172	12
H3	220	220	180	140	120	–	176	46
H4	200	200	220	220	150	150	190	32
H5	165	160	190	170	170	170	171	10
H6	135	100	145	130	135	125	128	15
H7	190	155	130	170	135	120	150	27
H8	165	130	200	200	200	180	179	28
H9	160	160	170	170	150	160	162	8
H10	140	130	170	175	150	150	153	17
H11	145	165	180	165	155	160	162	12
H12	160	180	160	255	210	190	193	36
H13	190	210	210	190	200	190	198	10
H14	185	190	180	185	180	150	178	14
H15	140	135	130	150	150	130	139	9
H16	190	180	180	165	200	210	188	16
H17	165	170	–	–	–	–	168	4
H18	155	180	190	160	170	155	168	14
H19	190	175	170	160	140	150	164	18
H20	120	170	200	190	110	113	151	41

Data represent means ± SE (n = 6). Statistical difference in ear length of colored sweet corn hybrids was shown in **Supplementary Table 5**.

## Discussion

The objective of this study was to develop novel diversely colored sweet corn lines using conventional breeding to introgress the kernel color traits from different colored non-sweet corn maize into *su1* and *sh2* sweet corn inbreds. Our breeding program led to the development of nine CS inbreds (five *su1* and four *sh2* types) which were ultimately used to produce 11 CS hybrids (eight *su1* and three *sh2* types). Yellow-orange, red, purple, and blue were the prevalent kernel colors in the CS lines as opposed to the original, yellow-colored parental sweet corns lines. The carotenoids were strongly concentrated in the sweet corn parents and the light-colored CS inbreds such as CS2, CS4, and CS8. Yellow sweet corn lines are associated with high carotenoids even before introgression of additional kernel colors. The resultant CS inbreds such as CS1, CS3, CS5, CS7, and CS9 strongly exhibited dark kernel colors including red, purple, and blue. These phenotypes are mostly associated with the flavonoids rather than the carotenoids; thus, explaining why the concentration of the carotenoid pigments was reduced in these dark-kernelled CS inbreds. With increased carotenoids concentrations in some CS inbreds, these varieties can now be assessed for rigorous nutritional studies on potential health benefits. Although, the functions of the carotenoids in humans remain uncertain (Mayne, 1996), several recent studies have shown that the carotenoids specifically β-carotene, which is the common carotene in plants, have preventative properties for some type of cancer including lung and colon cancer, improve glucose levels and control the glycemic state contributing to diabetes prevention, reduced cardiovascular diseases, cataracts, and age-related macular degeneration (Mayne, 1996; Abdul-Hamid and Moustafa, 2014**;** Jain et al., 2006; Kim, 2016). Besides the carotenoids, the majority of the CS inbreds and hybrids exhibited dark kernel pigments with mostly purple, red, and blue hues (**Figure 2**; **Supplementary Figures 1**, **S2**). These pigments belong to the flavonoid group, specifically within the anthocyanin family (Žilić et al., 2012). Though less stable than the carotenoids, the anthocyanins exhibit similar health benefits such as degenerative disease prevention due to their ability to protect cell against oxidative damages (Leong et al., 2018). Future nutritional studies on selected colored sweet corn hybrids can follow up on the specific health benefits relating to specific anthocyanins and the effects of sweet corn cooking methodology and duration.

Phenolic acids were also identified due to their similarity to carotenoids and flavonoids in terms of antioxidant properties and association with kernel pigments, phenolic acids were reduced in nearly all CS inbreds except CS3. In fact, nearly all phenolic acids were increased in CS3. The reason for this trend is not known other than that CS3 was the only CS inbred that exhibited blue pigments. These results contradict the findings reported by Lago et al. (2014) in which higher amounts of phenolic acids in colored sweet corn were measured compared with the uncolored parental line. However, the results agree with Žilić et al. (2012) who reported that some multicolored maize lines had the lowest phenolic content but higher in the light and dark blue maize lines. In addition to reduced phenolic acids, there was also a reduction of some carotenoids in the CS inbreds with yellow orange phenotype. Results indicated that α-carotene, β-carotene, and β-cryptoxanthin decreased remarkably in CS8 inbred by 58.91%, 46.08%, and 265.87%, respectively. The cause for this reduction is not known. One study reported that the presence of the *se1* allele in sweet corn were correlated with the lower levels of the carotenoids (Branch et al., 2024). However, CS8 inbred has a *sh2* allele which may or may not have similar effects on carotenoid as *se1*. The instability of the carotenoids may also explain the reported decreases in concentrations in some CS inbreds. Carotenoids are highly unsaturated which make them unstable; therefore, substantially declining in biofortified orange maize during post-harvest processing and storage (Nkhata, 2020). Heat and moisture treatments have been reported to degrade carotenoid and phenolic acid contents in yellow-orange maize (Beta and Hwang, 2018). The freshly frozen CS samples were immediately lyophilized without thawing and moisture was kept minimal at all times during sample preparation. We cannot conclude as to why some CS inbreds had substantial increases and decreases of some pigment concentrations. The variation of the pigments in CS lines may be attributed to early harvesting of the sweet corn. Studies have shown that the kernel maturity may dictate the concentration of the carotenoids and flavonoids pigments; where the concentration of zeaxanthin and anthocyanins increased as the kernels became more mature (O’Hare et al., 2015; Hong et al., 2020). It was shown that the anthocyanin pigments started accumulating in purple waxy maize as early as 10–12 DAP but the peak pigment accumulation occurred from 24–29 DAP (Kim et al., 2020). In this study, the CS lines were harvested at 20 DAP which is a likely before the peak accumulation of pigments. Despite the fact that some CS lines are clearly in the earlier stages of pigment accumulation at 20 DAP, the variety of color increases the visual diversity and may ultimately increase the antioxidant properties of the end product.

Despite the increment and reduction of pigments concentrations found in CS lines, several limitations are noted. We observed unusual variation among the pigment concentrations in both parental and colored sweet corn lines (**Figures 5**–**7**; **Supplementary Figures 5**, **S8**). It is possible that the methods used were not optimized for detecting these pigments in maize. Furthermore, the number of biological replicates (n=3) was limited which would explain the reported high standard deviation across multiple parental and colored sweet corn. Most importantly, the selection for kernel palatability in CS lines may have also limited the selection of CS lines with strong kernel pigmentation. It was reported that kernel texture became tougher with maturity leading to diminished eating quality (O’Hare et al., 2015). In our breeding program, we selected for vivid kernel color phenotypes at the early stage of 20 DAP to ensure acceptable kernel sweetness and tenderness. Maintaining kernel color, sweetness, and tenderness was the priority throughout the development of the CS inbreds and hybrids. In early generations, many lines displayed beautiful and diverse rainbow colors at 20 DAP, but they were eventually discarded because of unsatisfactory kernel sweetness and texture. In contrast, some CS lines had favorable palatability qualities but lacked sufficient kernel color at 20 DAP. Hong et al. (2020) suggested that delaying the harvest until 28 DAP would maximize the anthocyanin accumulation. While later harvested ears do indeed have more intense pigmentation, they invariably have tough, mealy and chewy textures and reduced overall eating quality. Given the slow adaptation of functional foods due to the perceived risk and uncertainty by the consumer (Dolgopolova et al., 2015), it was imperative in this study not to sacrifice expected eating qualities for uncertain pigment benefits.

At each generation, colored sweet corn lines were selected for kernel palatability qualities including sweetness and tenderness. Preliminary evaluation of the selected CS lines indicated desirable sweetness and kernel texture comparable to parental sweet corn lines (**Tables 3**, **4**). The preliminary sensory evaluations indicated that the kernel sweetness quality is highly correlated with the endosperm sucrose content (Ledenčan et al., 2022). However, the confirmatory assays revealed somewhat unexpected reduction of sucrose contents in CS inbreds and hybrids of *sh2* type (**Figure 3**; **Supplementary Figure 3**). In contrast, only one CS inbred of *sh2* type had significantly high starch contents compared with parental line and two hybrids had higher starch contents than one or both parental inbreds (**Figure 4**; **Supplementary Figure 4**). The variation in sugar contents from the parental inbreds were mostly pronounced in the CS hybrids of *sh2* rather than *su1* type. This observation may be attributed to the fact that the *sh2* lines accumulate higher sugar content than *su1* varieties (Hu et al., 2021). As a result, the *sh2* CS lines may have been more sensitive to the introduction of a high starch background from crossing to QPM dent corn lines. Given that the sucrose content is often inversely correlated to starch, the incremental starch profile in the CS lines may be attributable to decreased sucrose. The *su1* and *sh2* phenotypes dictated the differences among the CS hybrids in terms of sugar contents. No significant variations were observed in terms of overall average ear weight and size (**Supplementary Tables 4**, **S5**). However, the differences between *su1* and *sh2* mutations were undoubtedly observed during the CS hybrid development either in terms of general germination, maturities, and pollen health. The *sh2* sweet corn lines have lower early vigor/germination in cooler soils and require warmer soils than *su1* (Hassell et al., 2003; Ledenčan et al., 2022). Even in the controlled greenhouse environment where the CS hybrids were developed, half of the possible *sh2* CS hybrids were not recovered due to poor germination or different maturities of the *sh2* CS inbreds. It therefore appears that the variation among the CS inbreds and hybrids were, at least in part, attributable to the differences between *su1* and *sh2* mutations.

In this study, some crosses between *su1* and *sh2* CS inbreds were carried out to produce heterozygous Su1su1Sh2sh2 F_1_ CS hybrids. Since the *su1* and *sh2* mutations act recessively, kernels are heterozygous in the F1 and phenotypes of the F_1_ kernels were wild type. However, the sugar analysis and kernel palatability tests were carried on the F_2_ segregating ears derived from these hybrids. The F_2_ lines segregated 9/16 wild type and 7/16 sweet corn following Mendelian dihybrid cross model (Patwardhan, 2022). Sample F_2_ ears and kernels used for these test evaluations contained both wild type and sweet corn; therefore, the levels of sucrose and kernel palatability reported in these hybrids may be attributed to the 7/16 portion of kernels. The evaluations were performed on 20 DAP lines and at this stage wild type and sweet corn kernels cannot be differentiated apart phenotypically. Samples were extracted from random kernels and it is possible that the majority of the tested kernels exhibited the sweet corn traits given the levels of sucrose and kernel sweetness reported in the results. Prior to further tests, hybrids containing both *su1* and *sh2* require further section to recover homozygous *su1su1sh2sh2* genotypes.

This study has developed sweet corn lines with vivid diverse kernel colors at the sweet corn prime-eating stage. Though these CS lines preliminarily have similar kernel palatability qualities similar to normal sweet corn, the evaluation method used was limited in ways including small number of replicates, use of untrained testers, and lack of robust or fully structured comparisons between CS lines and the parental sweet corn. As such, the evaluation of the sweet corn eating qualities presented in this study is only preliminary. While multiple ears were tested by at least three testers, employing a fully structured, instructed and blind testing panel was beyond the scope of this initial study. Consequently, the results presented here provide initial insights into the performance of the CS inbreds and hybrids in terms of sweet corn qualities. Furthermore, the visual appeal and acceptability will require very extensive and well-designed consumer tests. Our preliminary results set up the need for future studies which include a large testing panels, increased replication, and holistic comparisons both CS inbreds/hybrids with respective parental sweet corn simultaneously. Furthermore, future studies will increase the number of replicates and use multi-location test trials to evaluate yield and disease related agronomics.

In summary, we developed different *su1* and *sh2* colored sweet corn using conventional breeding to explore the maize kernel color diversity and the associated carotenoid and flavonoid pigments. We demonstrated that the color diversity increased some carotenoids and flavonoids in most colored sweet corn inbreds. Our results suggest that the increased contents of carotenoid and flavonoid pigments may ultimately contribute to added nutritional health benefits and market potential for sweet corn. Additionally, the sweet corn developed maintains acceptable eating qualities such as kernel sweetness and tenderness comparable to parental sweet corn lines. However, the evaluation of these kernel eating qualities was arbitrary and intensive sensory studies are needed. Future research will also continue the selection and development of *su1su1sh2sh2* double mutant colored sweet corn. Overall, the findings of this study conclude that color phenotypes can be expressed in sweet corn kernels by its prime eating stage without compromising the sweet corn’s fresh eating qualities.

## Data Availability

The original contributions presented in the study are included in the article/**Supplementary Material**. Further inquiries can be directed to the corresponding author.

## Supplementary material

Supplementary Figure 1 Sweet corn parents (S1, NE-EDR *su1*; S2, NE-EDR *sh2*; S4, IA5125 *su1*) and colored sweet corn inbreds (CS2–CS9). CS2, NE-EDR *sh2/*Blue Indian; CS4, IA5125 x Bloody Butcher; CS5, (NE-EDR *sh2*/BI)//Seneca Red Stalker; CS6, Double Red Sweet corn//(NE-EDR*su1*/Blue India) CS7, (NE-EDR*sh2*/Blue Indian)//Double Red Sweet corn; CS8, EDRs*h2/*Bloody Butcher; CS9: EDR*su1*/Bloody Butcher.

Supplementary Figure 2 Colored sweet corn hybrids (H2–H20). H2, CS1 + CS6; H3, CS1 + CS8; H4, CS1 + CS9; H5, CS2 + CS4; H6, CS2 + CS6; H7, CS2 + CS7; H8, CS2 + CS8; H9, CS3 + CS4; H10, CS3 + CS6, H11, CS3 + CS7; H12, CS3 + CS8; H13, CS3 + CS9; H14, CS4 + CS5; H15, CS4 + CS6; H16, CS4 + CS8; H17, CS4 + CS9; H18, CS5 + CS9; H19, CS6 + CS9; H20, CS8 + CS9.

Supplementary Figure 3 Sucrose content (g/100g) in F_2_ lines of CS hybrids derived from crossing *su1* and *sh2* CS inbreds. All numbers represent means ± SE (n=4).

Supplementary Figure 4 Total starch (g/100g) in F_2_ lines of CS hybrids derived from crossing *su1* and *sh2* CS inbreds. All numbers represent means ± SE (n=4).

Supplementary Figure 5 Additional carotenoid compounds. Concentration of carotenoids in sweet corns and colored sweet corn inbreds. All numbers represent means ± SE (n=3).

Supplementary Figure 6 Additional flavonoid compounds. Concentration of flavonoids in sweet corns and colored sweet corn inbreds. All numbers represent means ± SE (n=3).

Supplementary Figure 7 Additional flavonoid compounds. Concentration of flavonoids in sweet corns and colored sweet corn inbreds. All numbers represent means ± SE (n=3).

Supplementary Figure 8 Additional phenolic acid compounds. Concentration of phenolic acids in sweet corns and colored sweet corn inbreds. All numbers represent means ± SE (n=3).

Supplementary Table 1 Parental sweet corn and colored maize lines.

Supplementary Table 2 S2 Eating quality scores in F_5_ colored sweet corn inbreds.

Supplementary Table 3 Color, sweetness, and texture scores for nine CS hybrids (containing both su1 and sh2) evaluated during Summer 2024. Freshly harvested ears (20 DAP) (n=3) were scored for color (1 = yellow/white, 5 = purple, blue, red…) sweetness (1 = bland, 5 = sweet) and texture (1 = tough, 5 = tender).

Supplementary Table 4 Comparison of ear weight among F_2_ colored sweet corn lines derived from the F_1_ colored sweet corn hybrids. *P*-values < 0.05 were considered significant.

Supplementary Table 5 Comparison of ear length among F_2_ colored sweet corn lines derived from the F_1_ colored sweet corn hybrids. *P*-values < 0.05 were considered significant.
