# Supplementary figures and images for: Breeding colored sweet corn for improved micronutrient content
Source: Front Plant Sci. 2026 May 18;17:1813937. doi: 10.3389/fpls.2026.1813937 (PMC13222991; doi:10.3389/fpls.2026.1813937)

**S1**

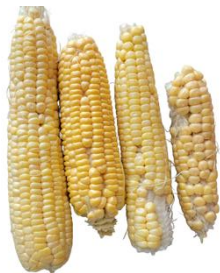

**CS6**

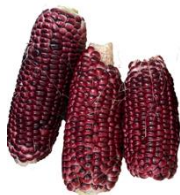

**CS9**

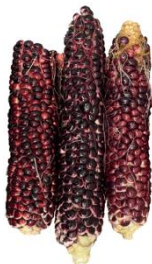

**S2**

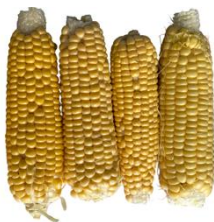

**CS2**

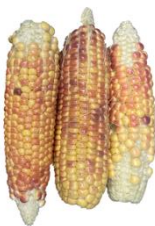

**CS5**

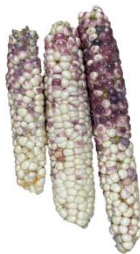

**CS7**

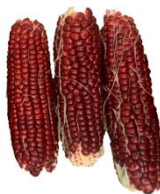

**CS8**

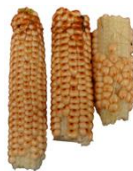

**S4**

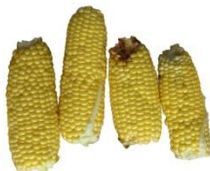

**CS4**

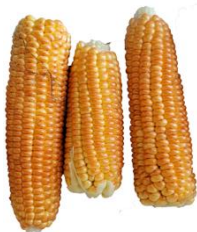

H2

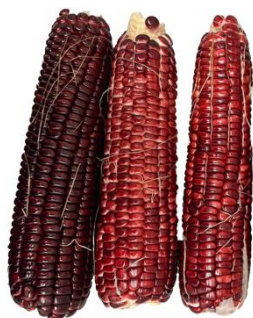

H3

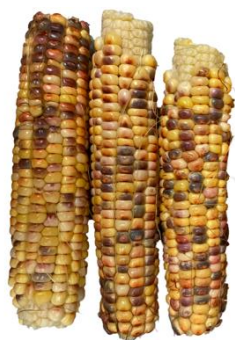

H4

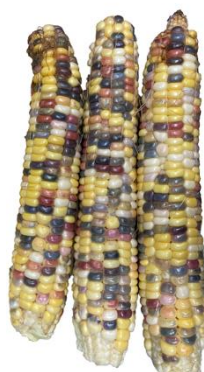

H5

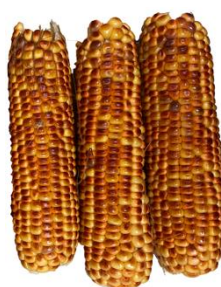

H6

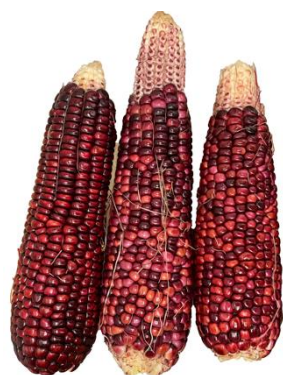

H7

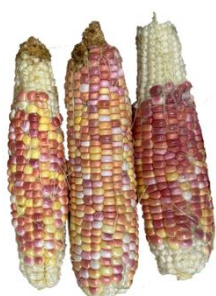

H8

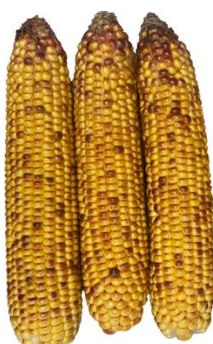

H9

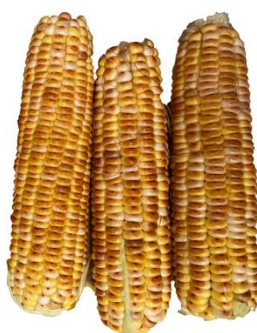

H10

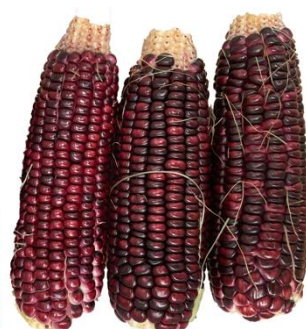

H11

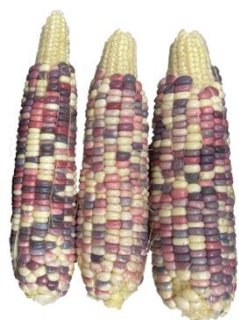

H12

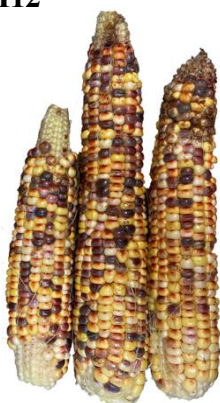

H13

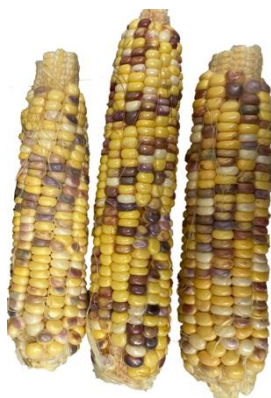

H14

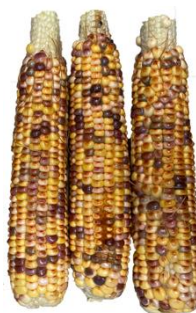

H15

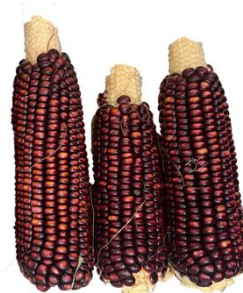

H16

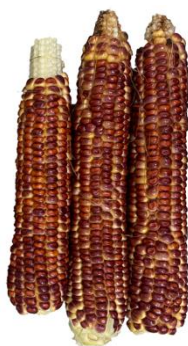

H17

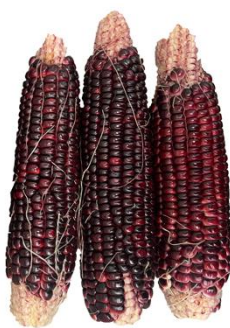

H18

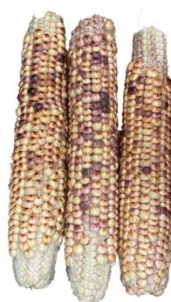

H19

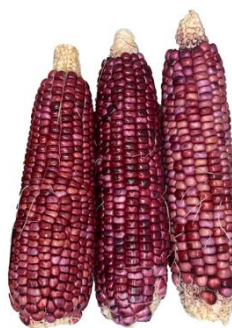

H20

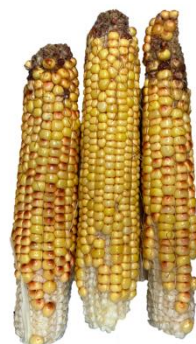

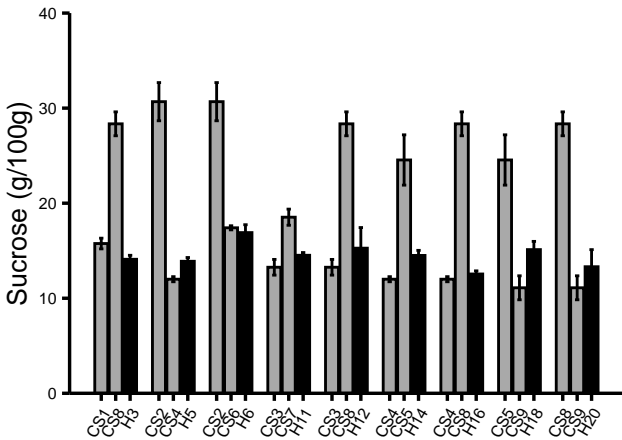

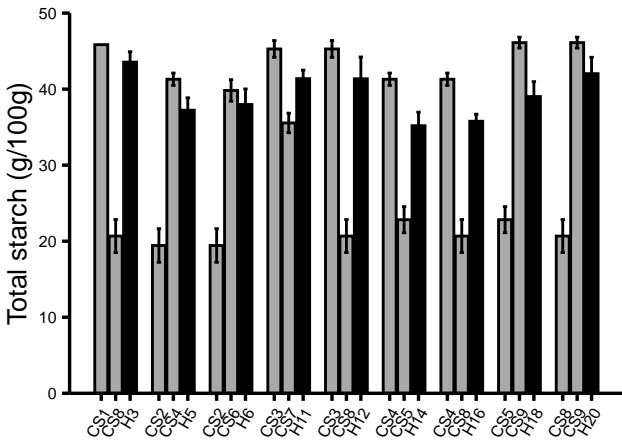

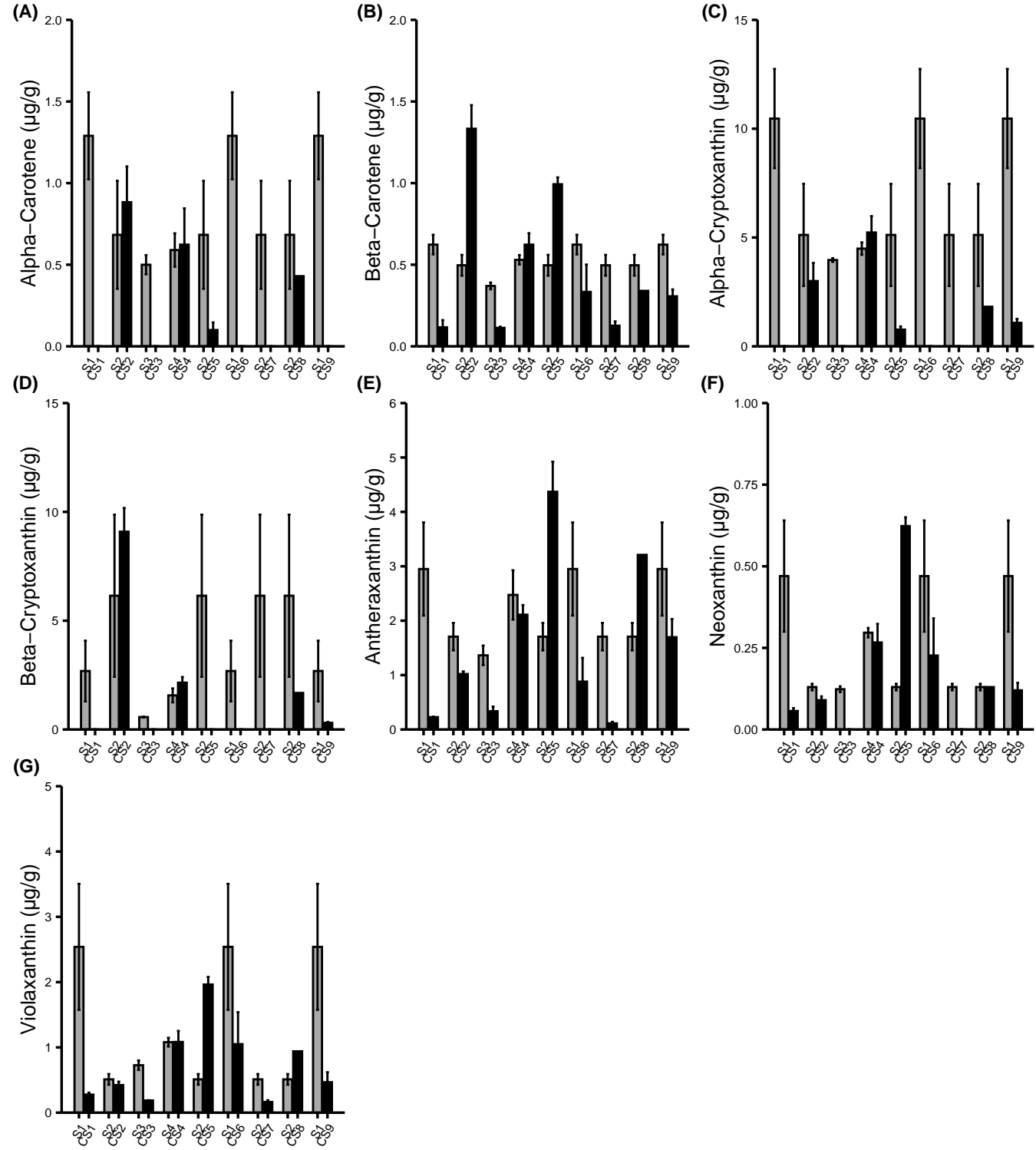

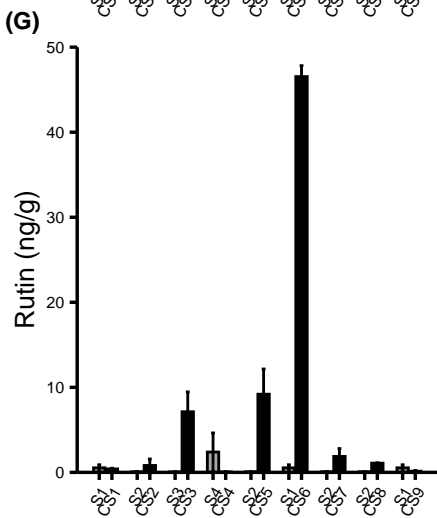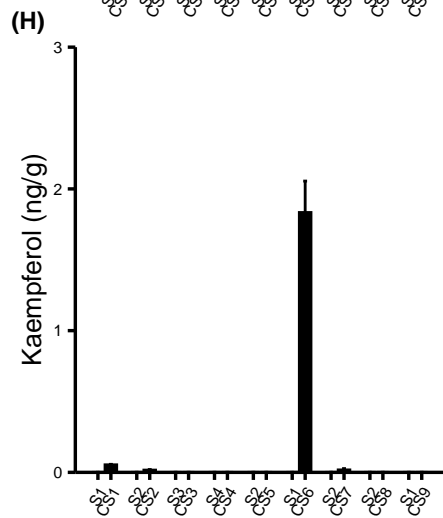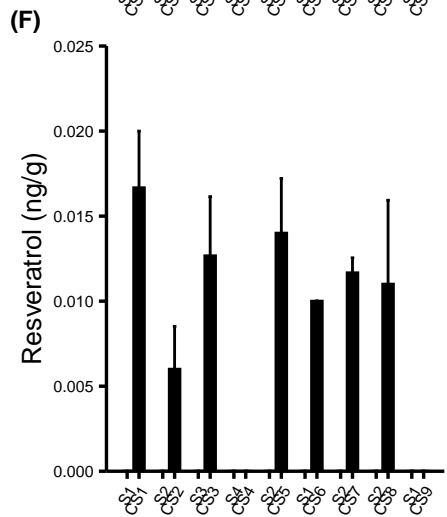

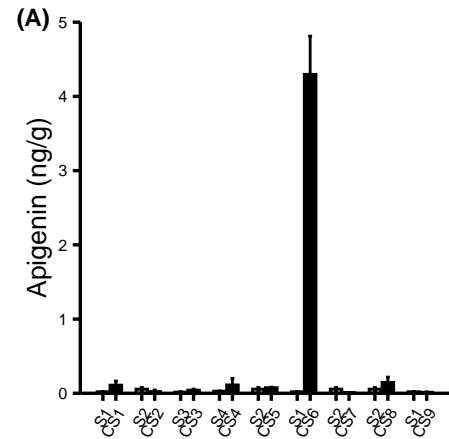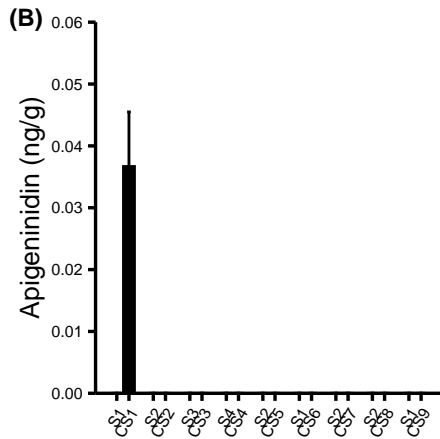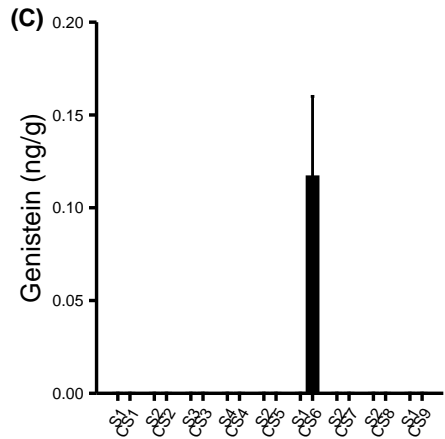

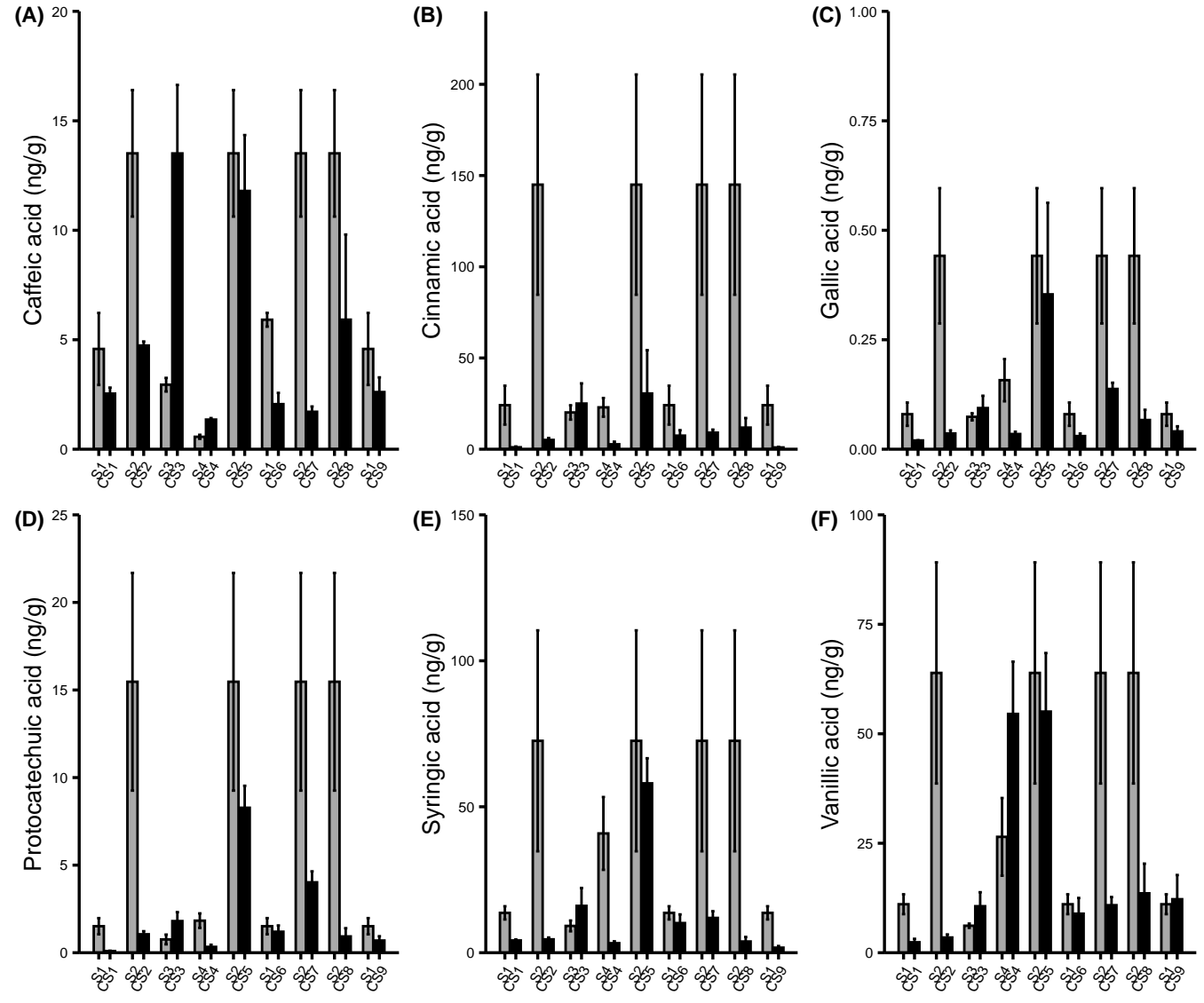

Supplement: Supplementary Figure 1 — Sweet corn parents (S1, NE-EDR su1; S2, NE-EDR sh2; S4, IA5125 su1) and colored sweet corn inbreds (CS2–CS9). CS2, NE-EDR sh2/Blue Indian; CS4, IA5125 x Bloody Butcher; CS5, (NE-EDR sh2/BI)//Seneca Red Stalker; CS6, Double Red Sweet corn//(NE-EDRsu1/Blue India) CS7, (NE-EDRsh2/Blue Indian)//Double Red Sweet corn; CS8, EDRsh2/Bloody Butcher; CS9: EDRsu1/Bloody Butcher. [file Image1.pdf]
