# Supplementary material for: Breeding colored sweet corn for improved micronutrient content
Source: Front Plant Sci. 2026 May 18;17:1813937. doi: 10.3389/fpls.2026.1813937 (PMC13222991; doi:10.3389/fpls.2026.1813937)
Supplement: Supplementary Table 1 — Parental sweet corn and colored maize lines. [file Table1.pdf]

Overview of parental maize varieties			
**Type**	**Variety name**	**Source**	**Description**
**Sweet corn**	NE EDR *sh2*	PI 614830	*shrunken-2* sweet corn
	NE EDR *su1*	PI 619435	*sugary-1* sweet corn
	IA5125 *su1*	Ames 587135	*sugary-1* sweet corn used as male in Iowa chief hybrid
	IA453 *su1*	Ames 22030	*sugary-1* sweet corn used as female in Iowa chief hybrid
	FL32 *su1+sh2*	PI 644100	*shrunken-2* and *sugary-1* sweet corn
	FL56 *su1+sh2*	PI 644099	*shrunken-2* and *sugary-1* sweet corn
	P39 *su1*	PI 587133	*sugary-1* sweet corn inbred used as female in Golden cross hybrid
	P51 *su1*	Ames 22049	*sugary-1* sweet corn inbred used as male in Golden cross hybrid
	Double red sweet corn	Mary's Heirloom Seeds	
**Dent corn**	Bloody Butcher	NSL26560	Deep red kernels with boasting great agronomic traits.
	Bloody Butcher Dark	Baker Creek Heirloom Seeds	A darker variation of the original bloody butcher variety
	Bloody Butcher Light	Baker Creek Heirloom Seeds	A lighter variation of the original bloody butcher variety
**Flint corn**	Rainbow Flint Red	-	Ornamental flint corn. Light orange-red in color; ground into flour for further processing uses.
	Blue Indian	NSL26565	Blue flint corn. Usually grown in Mexico and Southern US

Block #	F5 Colored Sweet corn	Color/Sweetness/Texture score	Select Lines for Hybrid Production	Colored Sweet Corn (CS) Inbreds
1	Rainbow Flinf Red x NE-EDR su1	2- 2- 2	Discard	
2	Blue Indian x P51 su1	3.5- 5- 3	Discard	
3	NE-EDR su1 x Blue Indian	5- 5- 5	Select	CS1
4	Blue Indian x NE-EDR sh2	1- 5- 5	Discard	
5	NE-EDR sh2 x Blue Indian	5- 5- 5	Select	CS2
6	IA453 su1 x Bloody Butcher	3- 3- 4		
7	IA5125 su1 x Bloody Butcher	4- 3- 5	Select	CS4
8	NE-EDR sh2 x Bloody butcher	3- 5- 5	Select	CS8
9	IA453 x Rainbow Flint Red	2- 4- 4	Discard	
10	P39 su1 x Blue Indian	5- 4- 5	Select	CS3
11	Blue Indian x FL56 sh2	1- 4- 4	Discard	
12	IA453 x Rainbow Flint Red	1- 3.5- 3.5	Discard	
13	Rainbow Flint Red x P39 su1	3- 4- 2	Discard	
14	(NE-EDR sh2 x Blue India) x Seneca Red Stalker	4- 5- 5	Select	CS5
15	(NE-EDR sh2 x Blue Indian) x Double Red Sweet corn	3.5- 3- 2	Discard	
16	Double Red Sweet corn x Bloody Butcher	5- 3.5- 3.5	Discard	
17	Double Red Sweet corn x (NE-EDR su1 x Blue Indian)	5- 5- 5	Select	CS6
27	(NE-EDR sh2 x Blue Indian) x Double Red Sweet corn	5- 4- 5	Select	CS7
28	NE-EDR su1 x Bloody Butcher	5- 4- 4	Select	CS9

Palatability scores in F2 lines derived from F1 CS hybrids.

	Color	Sweetness	Texture
**H3**	4	6	6
**H5**	6	5	3
**H6**	6	6	3
**H11**	5	4	4
**H12**	4	4	4
**H14**	6	5	5
**H16**	5	5	5
**H18**	5	5	3
**H20**	5	6	4

Ear weight comparisons among F_2 lines derived from the F_1 CS Hybrids.

	H1	H2	H4	H7	H8	H9	H10	H13	H15	H17
**H1**										
**H2**	1.000									
**H4**	1.000	1.000								
**H7**	1.000	1.000	1.000							
**H8**	0.206	0.741	0.176	0.186						
**H9**	0.116	0.569	0.097	0.103	1.000					
**H10**	0.972	1.000	0.958	0.963	0.998	0.986				
**H13**	0.639	0.985	0.588	0.606	1.000	1.000	1.000			
**H15**	0.045	0.935	0.036	0.039	1.000	1.000	0.910	0.999		
**H17**	0.590	0.949	0.547	0.562	1.000	1.000	1.000	1.000	1.000	
**H19**	0.650	0.987	0.598	0.617	1.000	1.000	1.000	1.000	0.999	1.000

Highlighted p-values were less than 0.05 were considered significant.

Ear length comparisons among F_2 lines derived from the F_1 CS Hybrids.

	H1	H2	H4	H7	H8	H9	H10	H13	H15	H17
**H1**										
**H2**	1.000									
**H4**	1.000	0.997								
**H7**	0.904	0.981	0.223							
**H8**	1.000	1.000	1.000	0.774						
**H9**	1.000	1.000	0.812	1.000	0.998					
**H10**	0.960	0.995	0.328	1.000	0.877	1.000				
**H13**	0.972	0.877	1.000	0.043	0.995	0.369	0.074			
**H15**	0.369	0.597	0.024	1.000	0.223	0.972	1.000	0.003		
**H17**	1.000	1.000	0.998	1.000	1.000	1.000	1.000	0.938	0.952	
**H19**	1.000	1.000	0.904	1.000	1.000	1.000	1.000	0.503	0.926	1.000

Highlighted p-values were less than 0.05 were considered significant.
